# Targeting PFKFB4 Biomimetic Codelivery System Synergistically Enhances Ferroptosis to Suppress Small Cell Lung Cancer and Augments the Efficacy of Anti‐PD‐L1 Immunotherapy

**DOI:** 10.1002/advs.202417374

**Published:** 2025-04-11

**Authors:** Xiang Liu, Jingjun He, Haoxuan Ying, Cuiying Chen, Chongyang Zheng, Peng Luo, Weiliang Zhu, Ting Wei, Bufu Tang, Jian Zhang

**Affiliations:** ^1^ Department of Oncology Zhujiang Hospital, Southern Medical University Guangzhou Guangdong 510280 China; ^2^ Department of Radiation Oncology Zhongshan Hospital Affiliated to Fudan University Shanghai 200032 China

**Keywords:** ferroptosis, immunotherapy, PFKFB4, Small cell lung cancer, tumor immune microenvironment

## Abstract

Small cell lung cancer (SCLC) is an extremely aggressive and highly malignant type of lung cancer that frequently develops resistance and recurrence following initial treatment. Paclitaxel (PTX) is a second‐line therapeutic option for SCLC patients with resistance to first‐line treatment. However, its clinical application is limited due to suboptimal efficacy and the risk of hypersensitivity reactions. To address these challenges, a novel therapeutic strategy employing a cationic liposome‐based biomimetic drug co‐delivery system, siPFKFB4/PRL_PTX_@RBCM‐cRGD, which simultaneously delivers paclitaxel and PFKFB4‐targeting small interfering RNA (siRNA) to SCLC cells and tissues is proposed. These findings demonstrate that this co‐delivery system can induce ferroptosis in SCLC cells, thereby enhancing their sensitivity to paclitaxel. Moreover, It promotes the infiltration of immune cells and the secretion of cytokines within the SCLC immune microenvironment, effectively activating anti‐tumor immunity. When combined with anti‐PD‐L1 antibodies, it further potentiates anti‐tumor immune responses. These results suggest that the biomimetic codelivery system not only induces ferroptosis to enhance paclitaxel efficacy but also reprograms the SCLC immune microenvironment, thereby potentiating the effects of anti‐PD‐L1 immunotherapy and providing a promising new therapeutic strategy for SCLC.

## Graphical abstract

In this study, a targeting PFKFB4 biomimetic codelivery system is developed to improve paclitaxel efficacy by inducing ferroptosis in SCLC. This system enhances immune cell infiltration and cytokine secretion, activating anti‐tumor immunity. Combined with anti‐PD‐L1, it boosts immune responses, offering a novel therapeutic strategy for overcoming resistance and recurrence in small‐cell lung cancer.

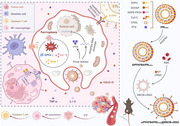

## 1 Introduction

Lung cancer remains the leading cause of cancer‐related mortality worldwide.^[^ ^1^ ^]^ Small cell lung cancer (SCLC), an aggressive subtype of pulmonary malignancy, is characterized by its high degree of malignancy and a propensity for metastasis at the time of diagnosis. Consequently, SCLC patients face significantly lower survival rates and greater therapeutic challenges compared to those with non‐small cell lung cancer (NSCLC).^[^ ^2^ ^]^ Currently, the standard first‐line treatment for SCLC consists of radiotherapy combined with platinum‐based doublet chemotherapy.^[^ ^3^ ^]^ Despite an initial high sensitivity to chemotherapy, SCLC frequently develops resistance and recurs, with a median overall survival (OS) of only 4–5 months following second‐line chemotherapy after relapse.^[^ ^4^ ^]^ Paclitaxel (PTX), as one of the second‐line therapeutic agents for SCLC, has demonstrated antitumor activity in refractory and relapsed SCLC cases.^[^ ^5^ ^]^ However, its efficacy as a monotherapy remains suboptimal. Furthermore, the use of solvent‐based formulations increases the risk of hypersensitivity reactions, thereby limiting its clinical application in second‐line treatment for SCLC.^[^ ^6^, ^7^ ^]^

In addition to chemotherapy, immune checkpoint inhibitors (ICIs) have been approved for the treatment of SCLC.^[^ ^8^ ^]^ However, only a limited subset of patients derives significant benefit from ICI therapy. The primary reason lies in the unique immune microenvironment of SCLC, which differs from other solid tumors and is characterized as a “cold” tumor immune microenvironment (TIME).^[^ ^9^ ^]^ Immunogenic cell death (ICD) is a form of cell death capable of activating adaptive immune responses.^[^ ^8^, ^10^ ^]^ In recent years, ferroptosis‐triggered immunogenic cell death has garnered significant attention.^[^ ^11^, ^12^ ^]^ The lipid peroxides generated during ferroptosis can induce the production of tumor‐associated antigens by tumor cells.^[^ ^13^ ^]^ Furthermore, ferroptosis promotes the release of damage‐associated molecular patterns (DAMPs), such as HMGB1 and ATP, through oxidative stress.^[^ ^14^ ^]^ These molecular signals recruit and activate dendritic cells (DCs), thereby initiating antigen presentation. Additionally, ferroptosis increases intracellular iron levels and reactive oxygen species (ROS) accumulation, triggering ICD and further modulating the tumor microenvironment.^[^ ^15^ ^]^

6‐Phosphofructo‐2‐kinase/fructose‐2,6‐bisphosphatase 4 (PFKFB4), a bifunctional enzyme of the PFKFB family, critically regulates glycolytic flux and autophagy. Numerous studies have reported the overexpression of PFKFB4 in various human cancers. It enhances chemosensitivity in SCLC by suppressing autophagy, positioning it as a therapeutic target for drug‐resistant SCLC.^[^ ^16^ ^]^ Beyond tumor metabolism, PFKFB4 modulates immune responses through glycolysis‐dependent cytokine regulation.^[^ ^17^ ^]^ Notably, a co‐delivery system combining rapamycin, anti‐PFKFB4 siRNA, and AS1411 aptamer demonstrated dual therapeutic and immunomodulatory effects, underscoring PFKFB4's potential in combinatorial cancer therapy.^[^ ^18^ ^]^ However, the relationship between PFKFB4 and the immune microenvironment of SCLC requires further investigation.

In summary, paclitaxel monotherapy demonstrates limited efficacy in the treatment of SCLC. Although novel paclitaxel formulations, such as paclitaxel liposomes, have reduced toxicity and hypersensitivity to some extent, their improvement in antitumor efficacy remains modest. PFKFB4 has shown potential as an antitumor target in enhancing chemosensitivity and modulating the immune microenvironment. Based on these findings, this study proposes a novel biomimetic drug delivery system designed to co‐deliver paclitaxel and PFKFB4‐targeting siRNA for the treatment of SCLC. Cationic liposomes were employed as nanocarriers, and their surface was coated with cRGD‐modified red blood cell membranes (RBCM) to enhance tumor‐targeting capability and achieve prolonged circulation in vivo. This biomimetic delivery system is expected to induce ferroptosis, improve SCLC sensitivity to paclitaxel, and activate immune responses, thereby enhancing antitumor efficacy and offering a new therapeutic strategy for SCLC.

## 2 Results

### 2.1 PFKFB4 is Associated with the Immune Microenvironment of SCLC and Enhances Paclitaxel Sensitivity

**Figure 1:**
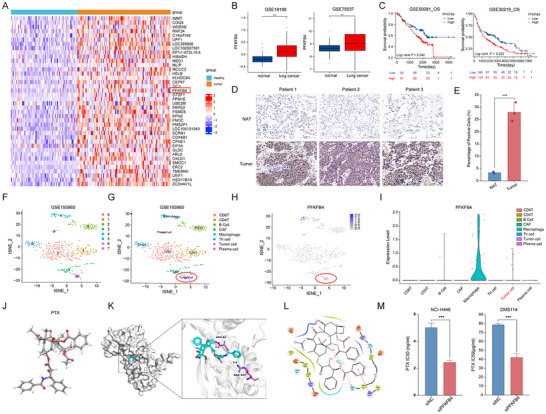
The Role of PFKFB4 in Small Cell Lung Cancer (SCLC). A) GEO database analysis of differentially expressed genes in lung cancer, with PFKFB4 highlighted in the red box. B) GEO lung cancer dataset analysis shows that the expression level of PFKFB4 in lung cancer is significantly higher than in normal tissue. C) GEO lung cancer dataset analysis reveals that higher expression of PFKFB4 correlates with poorer overall survival (OS) in lung cancer patients. D,E) Immunohistochemical analysis of PFKFB4 expression levels in adjacent non‐tumor and tumor tissues from small cell lung cancer (SCLC) patients, along with quantitative analysis. F,G) Single‐cell RNA sequencing analysis of the immune microenvironment in SCLC, identifying various cell types. H,I) Single‐cell analysis of PFKFB4 expression in CD8+ T cells and other cell types, with PFKFB4 predominantly expressed in macrophages. J) Schematic diagram of the 3D structure of paclitaxel. K,L) Docking of PFKFB4 with paclitaxel, shows that ASN64 of PFKFB4 interacts with paclitaxel via multiple forces, with a binding energy of −7.8 kcal mol^−1^. M) Comparison of the IC50 values of paclitaxel before and after knockdown of PFKFB4 in NCI‐H446 and DMS114 SCLC cell lines. ^*^ *p* < 0.05, ^**^ *p* < 0.01, ^***^ *p* < 0.001.

To investigate the expression of PFKFB4 in lung cancer, we analyzed data from the GEO and TCGA databases. As shown in **Figure** **1**A,B and S1A,B (Supporting Information), PFKFB4 expression was significantly higher in lung cancer tissues compared to normal tissues. Kaplan–Meier (KM) survival curves from the GSE50081 and GSE30219 datasets indicated that SCLC patients with high PFKFB4 expression have a poorer prognosis (Figure 1C).

To further determine PFKFB4 expression in SCLC, we collected tumor and adjacent non‐tumor tissues from three SCLC patients and analyzed PFKFB4 protein expression using IHC (Figure 1D). Quantitative analyses (Figure 1E) showed that PFKFB4 was highly expressed in SCLC tissues. Next, we annotated and clustered various cell populations, including T cells (Figure 1F,G), using the GSE193960 single‐cell SCLC dataset. We further annotated tumor cells using four markers closely associated with neuroendocrine tumors: ASCL1, NCAM1, INSM1, and GRP (Figure S1H, Supporting Information). These tumor cells are highlighted with red circles in Figure 1G,H. The results in Figure 1H,I show that PFKFB4 is expressed in macrophages, tumor cells, and B cells, with the highest expression observed in macrophages. In addition, validation using TCGA datasets revealed a negative correlation between PFKFB4 and multiple immune cell types, including CD8+ T cells, macrophages, and dendritic cells (Figure S1C—F, Supporting Information). These findings suggest that PFKFB4 has multiple roles in small‐cell lung cancer, including involvement in tumorigenesis and the formation of an immunosuppressive microenvironment.

Paclitaxel is one of the second‐line treatments for SCLC. Figure 1J shows the 3D structure of paclitaxel. Molecular docking analysis revealed multiple interactions between PFKFB4 and paclitaxel, such as a hydrogen bond between ASN64 of PFKFB4 and the ligand. These interactions resulted in a binding energy of −7.8 kcal mol^−1^ for the protein‐ligand complex (Figure 1K,L).To verify whether PFKFB4 affects paclitaxel sensitivity in SCLC, we knocked down PFKFB4 in NCI‐H446 and DMS114 SCLC cell lines (Figure S2A—C, Supporting Information). Subsequent IC50 assays showed that the IC50 value of paclitaxel in NCI‐H446 cells decreased from 5.01 to 2.43 ng mL^−1^, and in DMS114 cells from 78.57 to 41.91 µg mL^−1^ (Figure 1M). These results indicate that knockdown of PFKFB4 significantly enhances SCLC sensitivity to paclitaxel (*p* < 0.05).

### 2.2 Modified Nanoparticles Stably and Efficiently Delivers siPFKFB4 while Actively Targeting SCLC

**Figure 2:**
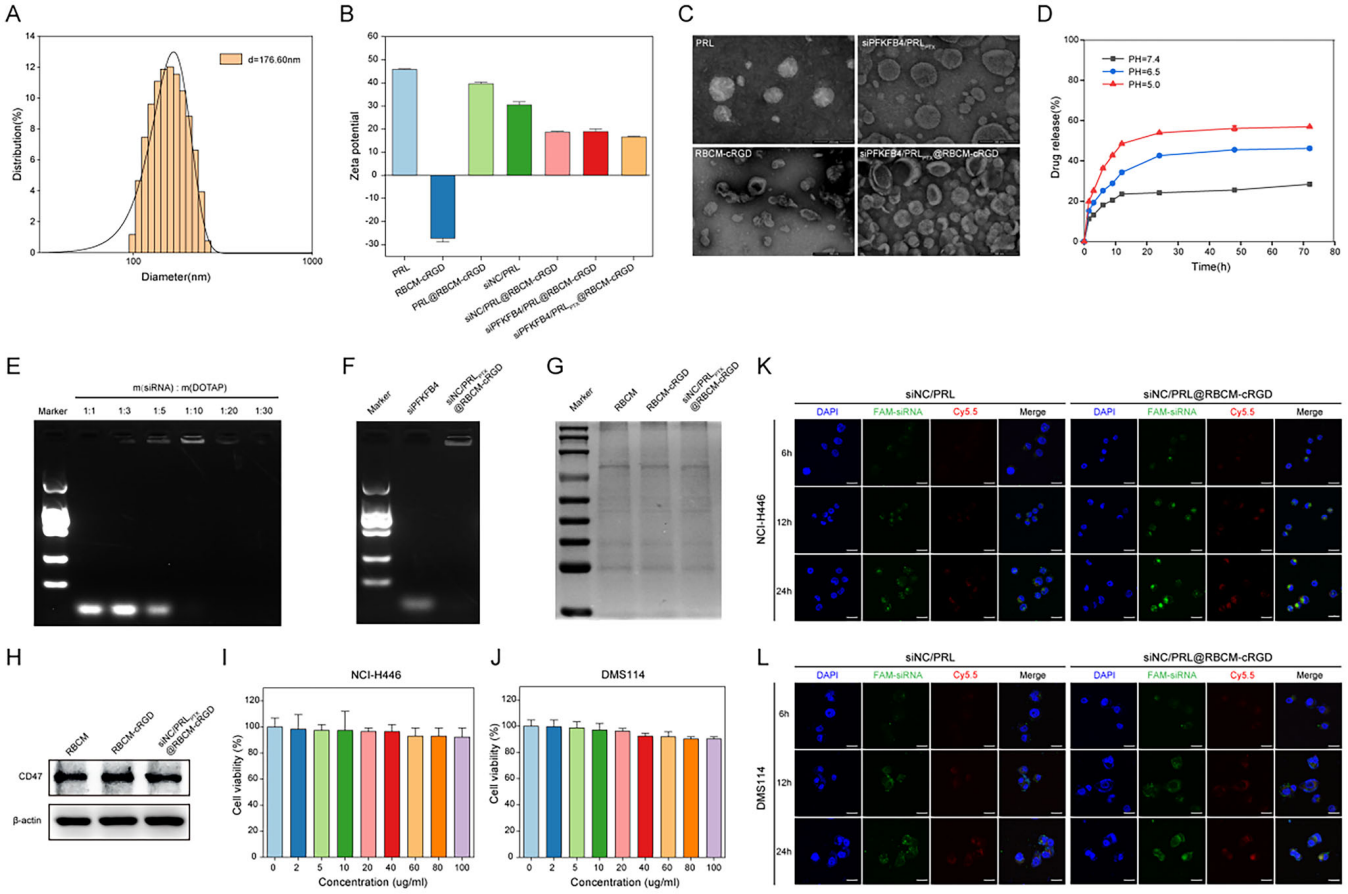
Synthesis and Characterization of siPFKFB4/PRLPTX@RBCM‐cRGD. A) Particle size distribution of siPFKFB4/PRL_PTX_@RBCM‐cRGD. B) Zeta potential of red blood cell membrane vesicles, siRNA, and various nanoparticles. C) Electron microscopy images (200 nm) of empty liposome PRL, PTX‐loaded liposome PRLPTX, RBCM‐cRGD, and siPFKFB4/PRL_PTX_@RBCM‐cRGD. D) In vitro release rates of paclitaxel under different pH conditions (pH = 5.0, pH = 6.5, pH = 7.4). E) Gel retardation assay showing the binding of siRNA with cationic liposomes at different mass ratios. F) Agarose gel electrophoresis results indicate complete binding of siRNA and cationic liposomes in siPFKFB4/PRL_PTX_@RBCM‐cRGD. G,H) SDS‐PAGE and Western blot analysis of protein retention and CD47 expression in RBCM, RBCM‐cRGD, and siPFKFB4/PRL_PTX_@RBCM‐cRGD. I,J) Cytotoxicity of siPFKFB4/PRL_PTX_@RBCM‐cRGD in NCI‐H446 and DMS114 SCLC cell lines. K,L) Uptake of siNC/PRL and siNC/PRL@RBCM‐cRGD in NCI‐H446 and DMS114 cells at different time points (6, 12, 24 h). Scale bars: 50 µm.

Cationic liposomes were synthesized using a thin‐film dispersion method, where DOTAP was utilized to confer a positive charge to PRL, enabling siRNA loading via electrostatic interactions. Red blood cell membranes (RBCM) were extracted using hypotonic lysis, followed by co‐extrusion and ultrasonic homogenization to obtain cRGD‐modified RBC vesicles. Finally, co‐extrusion of the liposomes and vesicles resulted in the siPFKFB4/PRL_PTX_@RBCM‐cRGD nanoparticles. The images, particle sizes, and zeta potentials of all nanoparticles used in this study are presented in Figure S3 (Supporting Information) and **Figure** **2**B. The siPFKFB4/PRL_PTX_@RBCM‐cRGD nanoparticles exhibited a particle size of ≈176.60 nm and a zeta potential of 16.6 mV (Figure 2A,B). The successful encapsulation of siRNA and RBC vesicles was confirmed by a significant decrease in zeta potential compared to blank nanoparticles (PRL) and other formulations. Figure 2C displays transmission electron microscopy (TEM) images of the blank liposomes (PRL), PTX‐loaded liposomes (PRL_PTX_), RBCM‐cRGD vesicles, and siPFKFB4/PRL_PTX_@RBCM‐cRGD nanoparticles. The pH‐responsive behavior of siPFKFB4/PRL_PTX_@RBCM‐cRGD was also evaluated. The drug release profile under simulated physiological (pH 7.4) and acidic (pH 6.5 and 5.0) conditions at 37 °C is shown in Figure 2D. The results indicated enhanced drug release in acidic environments, particularly at pH 5.0 (mimicking intracellular/lysosomal conditions), where the 48 h release rate reached 56.15 ± 1.22%.

**Figure 3:**
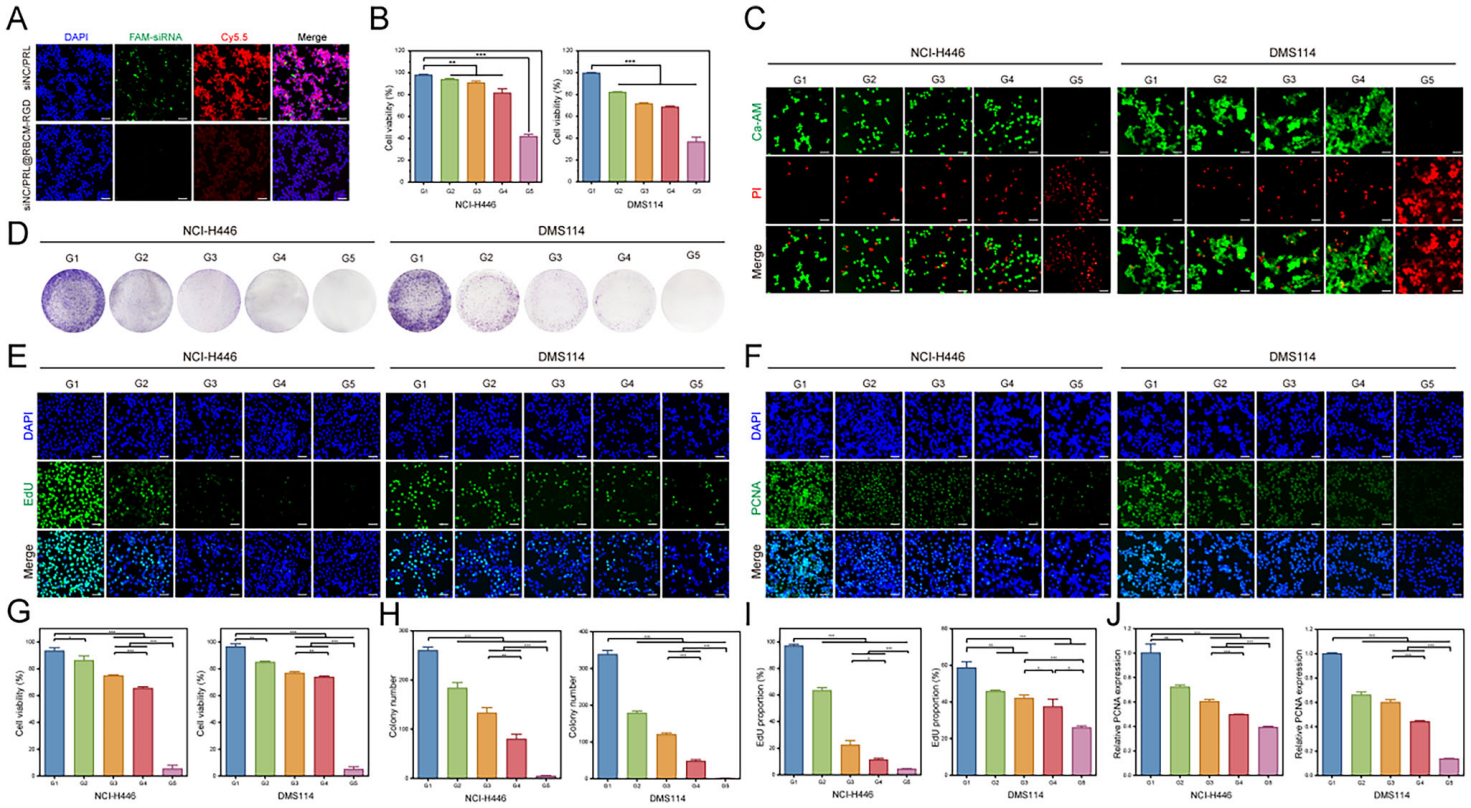
Antitumor Activity of The Biomimetic Codelivery System In Vitro. A) Uptake of siNC/PRL and siNC/PRL@RBCM‐cRGD by RAW264.7 cells after 24 h. B) CCK‐8 assay assessing the cell viability of NCI‐H446 and DMS114 SCLC cells in groups G1‐G5 (G1: PRL@RBCM‐cRGD, G2: PTX, G3: siNC/PRL@RBCM‐cRGD, G4: siPFKFB4/PRL@RBCM‐cRGD, G5: siPFKFB4/PRL_PTX_@RBCM‐cRGD). C) Live/dead cell staining of NCI‐H446 and DMS114 cells treated with G1‐G5 groups (magnification: 400×, scale bars: 50 µm.). D–F) Clonogenic assay, EdU incorporation assay, and PCNA immunofluorescence staining to evaluate the proliferative capacity of cells in G1‐G5 groups. G,H) Quantification of live/dead cell staining, clonogenic assay, EdU incorporation, and PCNA immunofluorescence (n = 3). The concentration of PTX was 2.5 ng mL^−1^ for NCI‐H446 and 35 µg mL^−1^ for DMS114. ^*^ *p* < 0.05, ^**^ *p* < 0.01, ^***^ *p* < 0.001.

To optimize siRNA loading onto cationic liposomes, a gel retardation assay was performed to determine the optimal mass ratio. As shown in Figure 2E, complete binding was achieved at a 1:10 mass ratio, and successful siRNA encapsulation was further confirmed in Figure 2F. SDS‐PAGE analysis demonstrated that RBCM proteins were preserved during nanoparticle preparation (Figure 2G), while Western blot analysis confirmed the intact retention of CD47 protein (Figure 2H). The cytotoxicity of the nanoparticles was evaluated in NCI‐H446 and DMS114 SCLC cell lines using the CCK‐8 assay. Figure 2I,J show that cell viability remained above 85% at nanoparticle concentrations up to 100 µg mL^−1^, indicating high biocompatibility and biosafety. The tumor‐targeting capability of RBCM‐cRGD‐modified nanoparticles was assessed by comparing cellular uptake in SCLC cells. Over time, the fluorescence intensity of nanoparticles within cells increased (Figure 2K,L). At equivalent time points, siNC/PRL@RBCM‐cRGD demonstrated stronger intracellular fluorescence compared to siNC/PRL, confirming that RBCM‐cRGD enhanced tumor‐targeting capacity. Additionally, the uptake of nanoparticles by RAW264.7 macrophages was evaluated. **Figure** **3**A shows that siNC/PRL@RBCM‐cRGD was less phagocytosed by macrophages than siNC/PRL, further demonstrating that RBCM‐cRGD reduced macrophage‐mediated clearance and enabled prolonged circulation in vivo. This feature ensures the nanoparticles' functionality within the tumor microenvironment, enhancing their therapeutic potential.

To further investigate the colloidal stability of positively charged nanoparticles under physiological conditions, we conducted in vitro stability assessments in simulated serum environments over a 7‐day period. Dynamic light scattering analysis revealed that the nanoparticles maintained their structural integrity in both phosphate‐buffered saline (PBS) and fetal bovine serum (FBS), with no statistically significant variation in hydrodynamic diameter observed throughout the experimental duration. As demonstrated in Figure S3 (Supporting Information), the nanoparticle size distribution remained stable regardless of the dispersion medium, suggesting effective resistance against protein adsorption and aggregation in biological fluids. This sustained stability profile in serum‐containing conditions indicates promising potential for systemic circulation applications.

### 2.3 The Biomimetic Codelivery System Demonstrates Potent Cytotoxic Activity against SCLC

To evaluate the antitumor efficacy of the biomimetic codelivery system, we conducted functional assays on two SCLC cell lines, NCI‐H446 and DMS114. The CCK‐8 assay and live‐dead staining were first used to assess cell viability and cytotoxicity. Results from Figure 3B revealed a significant reduction in cell viability in the siPFKFB4/PRL_PTX_@RBCM‐cRGD treatment group compared to other groups. Live‐dead staining further confirmed that siPFKFB4/PRL_PTX_@RBCM‐cRGD exhibited stronger cytotoxicity than PTX alone(Figure 3C,G). To investigate cell proliferation, we performed colony formation, EdU, and PCNA immunofluorescence assays across the G1‐G5 groups. Consistently, the experimental results and quantitative analyses demonstrated that the biomimetic codelivery system effectively suppressed SCLC cell proliferation (Figure 3D–F,H–J). These findings indicate that the biomimetic codelivery system exerts strong in vitro tumor‐killing effects.

### 2.4 Co‐Delivery of PTX and siPFKFB4 via Nanocarriers Amplifies Ferroptosis in SCLC

**Figure 4:**
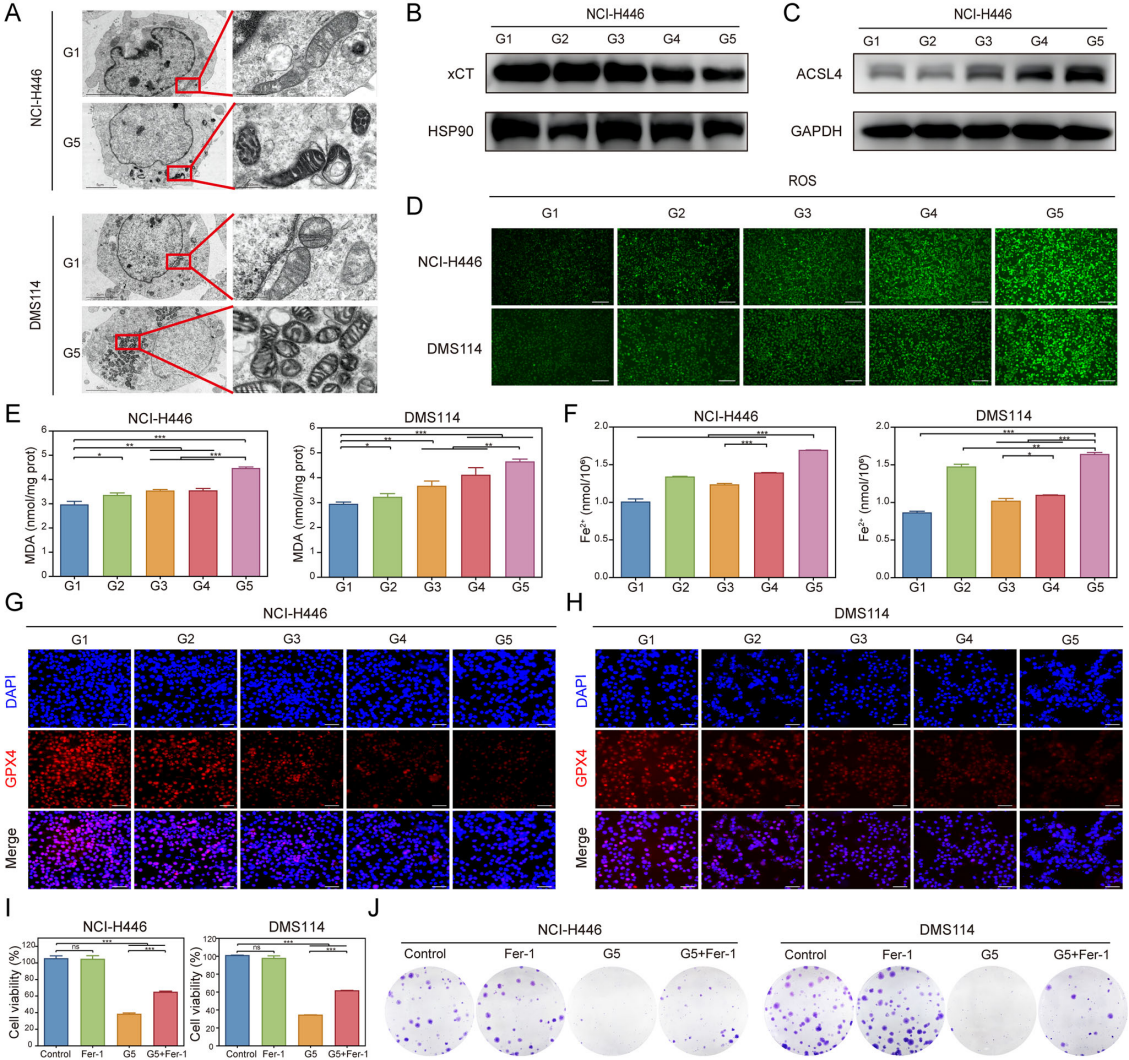
Synergistic Amplification of Ferroptosis by The Biomimetic Codelivery System to Inhibit SCLC. A) Electron microscopy images of NCI‐H446 and DMS114 cells treated with G1 and G5 group nanoparticles (left: 5 µm, right: 500 nm). B,C) Western blot analysis of xCT and ACSL4 expression in NCI‐H446 cells (internal reference: HSP90 and GAPDH). D) Intracellular ROS levels in NCI‐H446 and DMS114 cells treated with different groups. E,F) Intracellular levels of MDA and iron ions in NCI‐H446 and DMS114 cells under different treatments (n = 3). ^*^ *p* < 0.05, ^**^ *p* < 0.01, ^***^ *p* < 0.001. G,H) Immunofluorescence detection of GPX4 expression in NCI‐H446 and DMS114 cells in G1‐G5 groups. I) The CCK‐8 assay was employed to assess the impact of the combined treatment with Fer‐1 and siPFKFB4/PRL_PTX_@RBCM‐cRGD on the proliferative capacity of SCLC cells. J) The colony formation assay was performed to evaluate the anti‐proliferative effects of the combined treatment with Fer‐1 and the co‐delivery system in SCLC cells.

The superior antitumor efficacy of the co‐delivery of PTX and siPFKFB4 compared to PTX alone prompted further mechanistic investigations. Transmission electron microscopy revealed mitochondrial shrinkage and reduced mitochondrial cristae in G5‐treated cells, hallmark features of ferroptosis (**Figure** **4**A). Next, we assessed the expression levels of ferroptosis‐related proteins in the treated cell groups. Western blot analysis showed that siPFKFB4/PRL_PTX_@RBCM‐cRGD treatment significantly reduced xCT levels and upregulated ACSL4 expression in both cell lines (Figure 4B,C; Figure S4A,B, Supporting Information). GPX4 immunofluorescence staining and quantitative analysis indicated that GPX4 expression was markedly elevated in the G5 group compared to other groups (*p*< 0.05, Figure 4G,H; Figure S5B, Supporting Information). Reactive oxygen species (ROS) levels were also significantly altered across treatment groups, with the highest ROS levels observed in cells treated with siPFKFB4/PRL_PTX_@RBCM‐cRGD (Figure 4D; Figure S5A, Supporting Information). Additionally, malondialdehyde (MDA) and Fe^2+^ levels were markedly increased in the G5 group compared to the G2 group (Figure 4E,F). These results collectively demonstrate that the co‐delivery of PTX and siPFKFB4 promotes ferroptosis in SCLC cells, thereby enhancing their sensitivity to paclitaxel. To further clarify the relationship between the co‐delivery system and ferroptosis, we treated SCLC cells with a combination of the ferroptosis inhibitor Fer‐1 and the co‐delivery system, observing the changes in cell proliferation. The results presented in Figure 4I,J reveal that both the CCK‐8 and colony formation assays indicate that Fer‐1 alone does not affect the proliferation capacity of SCLC cells. However, when combined with the co‐delivery system (G5 group), Fer‐1 partially mitigates the inhibitory effect of the co‐delivery system on SCLC proliferation. These findings suggest that the co‐delivery system indeed enhances the role of ferroptosis in SCLC cells.

### 2.5 Nanoparticle Targeting of PFKFB4 Promotes M1 Polarization and Activates DC‐Induced Immune Responses In Vitro

**Figure 5:**
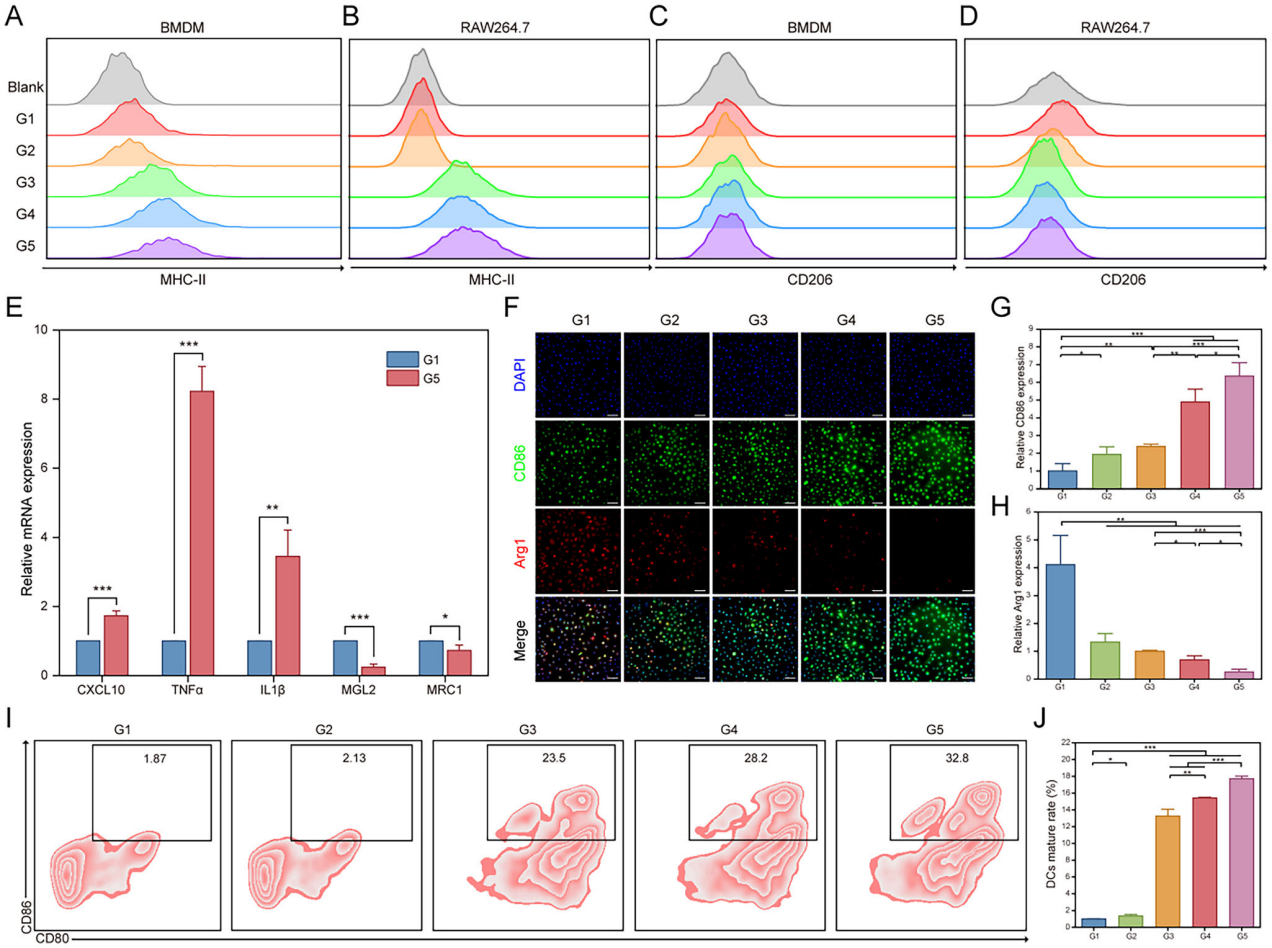
Status of Different Immune Cells after Co‐culture with siPFKFB4/PRL_PTX_@RBCM‐cRGD and RPM Cells. A,B) Flow cytometry analysis of MHC‐II expression, a marker for M1 polarization, in bone marrow‐derived macrophages (BMDM) and RAW264.7 cells after co‐culture. C,D) Flow cytometry analysis of CD206 expression, a marker for M2 polarization, in BMDM and RAW264.7 cells following co‐culture. E) RT‐qPCR analysis of M1 polarization markers CXCL10, TNF‐α, IL‐1β, and M2 polarization markers MGL‐2 and MRC1 mRNA expression levels in BMDM after co‐culture (n = 3). ^*^ *p* < 0.05, ^**^ *p* < 0.01, ^***^ *p* < 0.001. F–H) Immunofluorescence analysis of Arg1 and CD86 expression in BMDM cells co‐cultured with different nanoparticles and RPM cells, with quantitative analysis (n = 3). I,J) Percentage and quantitative analysis of CD80 and CD86 double‐positive dendritic cells (DCs) in G1‐G5 co‐culture groups.

Previous results indicated that PFKFB4 expression is negatively correlated with the infiltration of most immune cells in SCLC. To validate this, we utilized immortalized murine macrophages (RAW264.7) and induced bone marrow‐derived macrophages (BMDM) and dendritic cells (BMDC). These cells were co‐cultured with nanoparticles and murine SCLC cells to simulate the tumor microenvironment and explore the role of the biomimetic codelivery system in immune modulation (Figure S6, Supporting Information). We first assessed markers of M1 and M2 macrophage polarization. Flow cytometry revealed that MHC‐II expression in BMDM and RAW264.7 cells increased progressively from G1 to G5 groups, while CD206 expression decreased, suggesting a trend toward M1 polarization (**Figure** **5**A–D; Figure S7, Supporting Information). RT‐qPCR results further confirmed this shift, as M1‐associated chemokine IL1β, CXCL10 and TNF‐α levels were significantly elevated in the siPFKFB4/PRL_PTX_@RBCM‐cRGD group, while M2‐associated markers MGL2, and MRC1 were significantly downregulated (*p* < 0.05, Figure 5E). Immunofluorescence analysis showed increased expression of CD86 and reduced ARG1 levels in the G5 group compared to other groups, further supporting M1 polarization (Figure 5G,H). Next, we evaluated the maturation of BMDCs after co‐culture via flow cytometry. The proportion of CD80 and CD86 double‐positive DCs increased significantly across G1‐G5 groups, with the highest levels observed in the G5 group (Figure 5I). These findings demonstrate that the biomimetic codelivery system strongly promotes DC maturation. Collectively, the results indicate that the nanoparticle‐mediated targeting of PFKFB4 is closely associated with the SCLC immune microenvironment and exhibits robust immune activation capabilities in vitro.

### 2.6 Nanoparticle Co‐Delivery System Activates the JAK‐STAT Pathway to Modulate the Immune Microenvironment in SCLC

**Figure 6:**
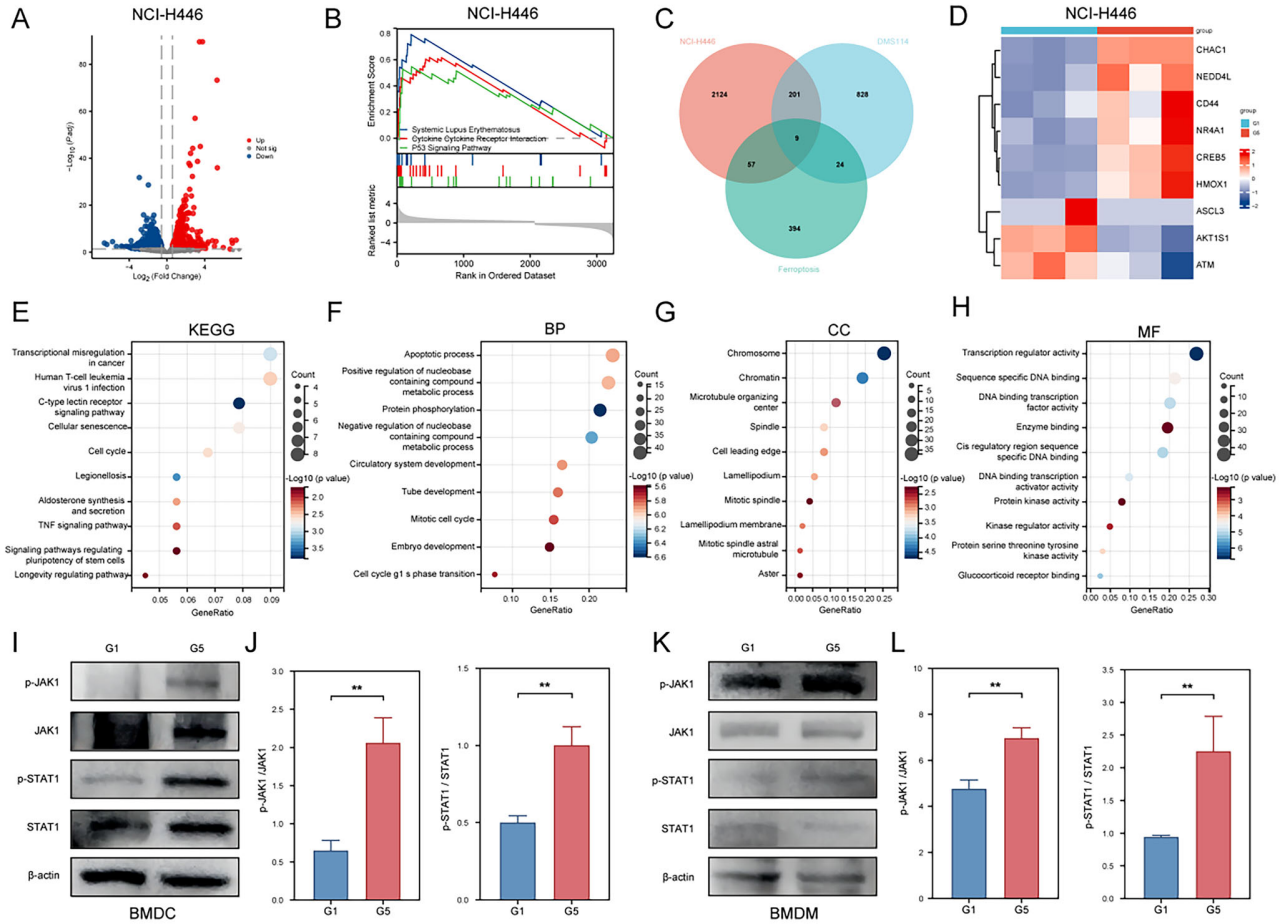
Mechanistic Exploration of The Biomimetic Codelivery System in Inhibiting SCLC. A) Volcano plot of differentially expressed genes after transcriptome sequencing of NCI‐H446 cells treated with RBCM‐cRGD and siPFKFB4/PRL_PTX_@RBCM‐cRGD. B) Gene Set Enrichment Analysis (GSEA). C) Venn diagram showing the intersection of differentially expressed genes in NCI‐H446 and DMS114 cells treated with siPFKFB4/PRL_PTX_@RBCM‐cRGD and ferroptosis‐related genes. D) Heatmap of the expression of 9 selected ferroptosis‐related genes in NCI‐H446 cells. E–H) KEGG and GO pathway enrichment analysis of the common differentially expressed genes between NCI‐H446 and DMS114 cells. I,J) Western blot analysis and quantitative analysis of JAK‐STAT pathway proteins in BMDC cells treated with RBCM‐cRGD and siPFKFB4/PRL_PTX_@RBCM‐cRGD (n = 3). K,L) Western blot analysis and quantitative analysis of JAK‐STAT pathway proteins in BMDM cells treated with RBCM‐cRGD and siPFKFB4/PRL_PTX_@RBCM‐cRGD (n = 3).

To elucidate the mechanisms underlying the antitumor effects of the nanoparticle co‐delivery system and its regulation of the SCLC immune microenvironment, we treated NCI‐H446 and DMS114 cells with PRL@RBCM‐cRGD and siPFKFB4/PRL_PTX_@RBCM‐cRGD, followed by transcriptomic analysis. Volcano plots highlighted differentially expressed genes (DEGs) after nanoparticle treatment (**Figure** **6**A; Figure S8A, Supporting Information). Gene Set Enrichment Analysis (GSEA) revealed enrichment of pathways associated with cytokine‐receptor interactions (Figure 6B; Figure S8B, Supporting Information). By intersecting DEGs with ferroptosis‐related genes, we identified nine genes potentially critical for ferroptosis induction by the system: ACSL3, AKT1S1, ATM, CD44, CHAC1, CREB5, HMOX1, NEDD4L, and NR4A1 (Figure 6C,D). Expression changes of these genes in the G1 and G5 groups were consistent with patterns observed during ferroptosis, further corroborating that the nanoparticle co‐delivery system enhances PTX sensitivity and antitumor efficacy by promoting ferroptosis. Functional enrichment analysis (Figure 6E–H) linked nanoparticle activity to cytokine‐receptor interactions and DNA‐binding pathways. Given the established role of the JAK‐STAT pathway in mediating cytokine effects,^[^ ^19^, ^20^ ^]^ we assessed the expression of key JAK‐STAT proteins in BMDCs and BMDMs co‐cultured with nanoparticles and SCLC cells. As shown in Figure 6I–L, expression of phosphorylated JAK1 (p‐JAK1) and phosphorylated STAT1 (p‐STAT1) was significantly elevated in immune cells after co‐culture, particularly in the siPFKFB4/PRL_PTX_@RBCM‐cRGD group. These findings suggest that JAK‐STAT pathway activation is a critical mechanism by which the biomimetic codelivery System modulates the SCLC immune microenvironment.

### 2.7 The Biomimetic Codelivery System Shows Excellent Targeting of SCLC Tumors with Minimal Toxicity

**Figure 7:**
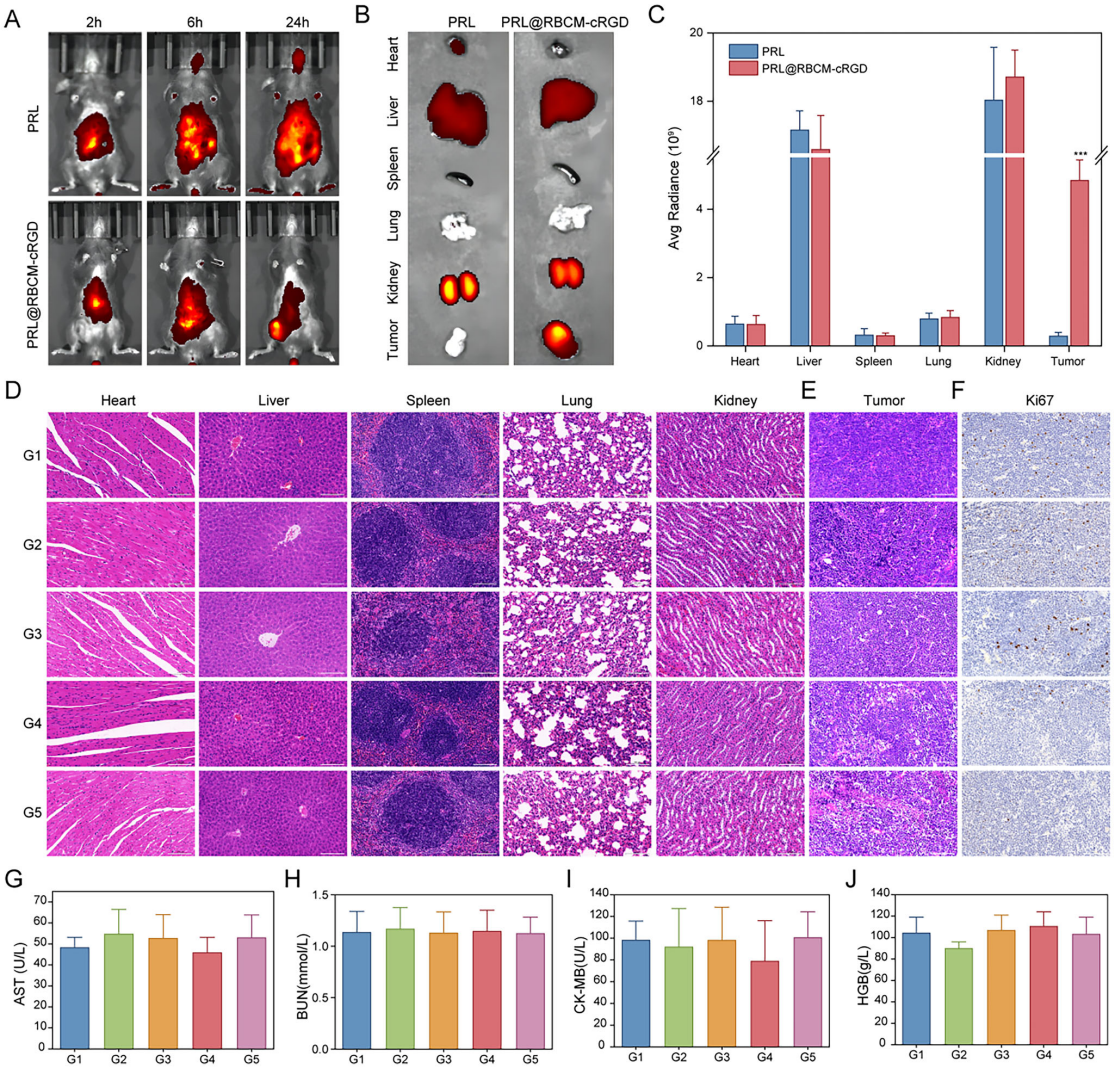
Biocompatibility and Tumor Targeting Ability of The Biomimetic Codelivery System in Vivo. A) In vivo imaging of C57 mice after subcutaneous tumor formation and tail vein injection of PRL and PRL@RBCM‐cRGD. Nanoparticle uptake was observed at 2, 12, and 24 h (n = 3). B,C) After 24 h of tail vein injection, mice were humanely euthanized, and imaging was performed on the heart, liver, spleen, lungs, kidneys, and tumor tissues to observe nanoparticle uptake, followed by quantitative analysis. D,E) Hematoxylin and eosin (H&E) staining of heart, liver, spleen, lungs, kidneys, and tumor tissues after tail vein injection of G1‐G5 nanoparticles or drugs (G1: PRL@RBCM‐cRGD, G2: PTX, G3: siNC/PRL@RBCM‐cRGD, G4: siPFKFB4/PRL@RBCM‐cRGD, G5: siPFKFB4/PRL_PTX_@RBCM‐cRGD). Scale bars: 100 µm. F) Immunohistochemical analysis of Ki67 expression in tumor tissues from G1‐G5 groups. G–J) Blood samples from G1‐G5 groups were collected to assess liver function (AST), kidney function (BUN), heart function (CK‐MB), and blood routine (HGB) (n = 3). ^*^ *p* < 0.05, ^**^ *p* < 0.01, ^***^ *p* < 0.001.

Given the promising antitumor efficacy and biosafety demonstrated by the biomimetic codelivery System in vitro, we proceeded to evaluate its therapeutic potential in vivo. A murine subcutaneous SCLC model was established using RPM cells. Mice were intravenously injected with Cy5.5‐labeled PRL or PRL@RBCM‐cRGD nanoparticles, and in vivo, fluorescence imaging was conducted at 2, 12, and 24 h to monitor nanoparticle distribution. As shown in **Figure** **7**A, both PRL and PRL@RBCM‐cRGD initially accumulated in the heart and liver but gradually localized to the tumor region over time. By 24 h, fluorescence signals were primarily concentrated in the liver, kidneys, and tumor sites, with PRL@RBCM‐cRGD showing significantly enhanced tumor accumulation compared to PRL (Figure 7B,C). These findings suggest that RBCM‐cRGD imparts enhanced tumor‐targeting capability to nanoparticles. To assess the biocompatibility of siPFKFB4/PRL_PTX_@RBCM‐cRGD, tumor‐bearing mice were injected with formulations identical to those used in vitro studies, and potential organ damage and hematological changes were evaluated. As shown in Figure 7D, neither PTX nor siPFKFB4/PRL_PTX_@RBCM‐cRGD caused significant organ damage. Liver, kidney, and cardiac functions, along with hematological indices, showed no notable differences among the five groups (Figure 7G–J; Figure S9B—F, Supporting Information), demonstrating the favorable biocompatibility of the biomimetic codelivery System.

### 2.8 Potent In Vivo Antitumor Efficacy of the Nanoparticle Co‐Delivery System Against SCLC

**Figure 8:**
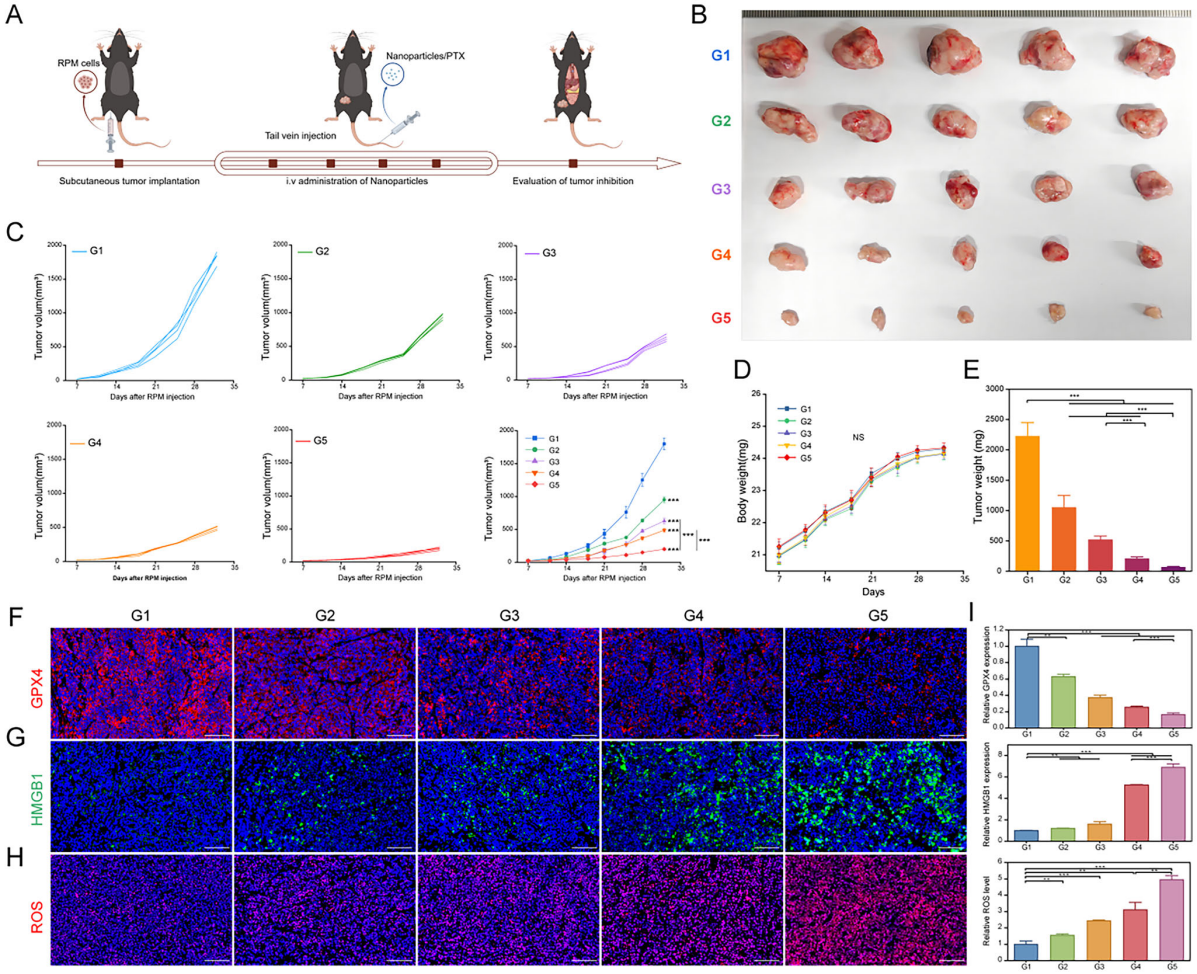
In Vivo Inhibition of SCLC by The Biomimetic Codelivery System. A) Treatment strategy for RPM tumor‐bearing mice. B) Tumor growth differences in SCLC after treatment with G1‐G5 nanoparticles (n = 5 per group). C) Tumor volume differences in mice across different treatment groups. D) Body weight changes in mice during the treatment period. E) Tumor weight at the end of treatment in different groups. F–I) Immunofluorescence analysis of GPX4, HMGB1 expression, ROS levels, and quantitative analysis in different treatment groups. ^*^ *p* < 0.05, ^**^ *p* < 0.01, ^***^ *p* < 0.001.

The in vivo antitumor efficacy of the nanoparticle co‐delivery system was subsequently evaluated. Tumor‐bearing mice were divided into five groups for further experiments, as outlined in **Figure** **8**A. PTX was administered at 10 mg kg^−1^ weekly for a total of four doses. Tumor volume was significantly larger in Group G1 compared to Group G5, where tumor volume was smallest (Figure 8B). Although PTX monotherapy reduced tumor size, the nanoparticle co‐delivery system exhibited superior antitumor efficacy (Figure 8C,E). Mouse body weight remained stable across all groups during treatment (Figure 8D), indicating the therapeutic regimen's safety. These results highlight the enhanced efficacy of combining siPFKFB4 with PTX in SCLC treatment. Post‐treatment tumor tissues were subjected to histological examination via hematoxylin‐eosin (HE) staining and Ki67 immunohistochemistry. HE staining revealed progressive increases in tumor necrosis and intercellular gaps from G1 to G5 (Figure 7E). Ki67 expression, an indicator of proliferation, declined progressively, with the lowest levels observed in G5 (Figure 7F; Figure S9A, Supporting Information). Immunofluorescence analysis further confirmed these findings; siPFKFB4/PRL_PTX_@RBCM‐cRGD‐treated tumors exhibited significant reductions in GPX4 expression and elevated reactive oxygen species (ROS) levels (Figure 8F,H), consistent with in vitro observations. These results suggest that the biomimetic codelivery system induces ferroptosis in SCLC cells.

### 2.9 PFKFB4‐Targeted Nanoparticle Co‐Delivery System: Immune Activation and Synergy with PD‐L1 Antibodies In Vivo

**Figure 9:**
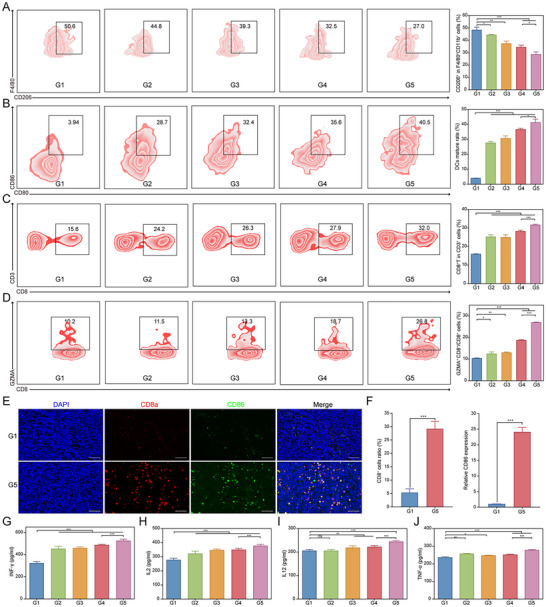
Tumor Immune Response Induced by The Biomimetic Codelivery System. A) Flow cytometry analysis of the proportion of M2 macrophages in tumor tissues from G1‐G5 groups and quantitative analysis. B) Differences in the maturation of dendritic cells (DCs) in tumor tissues from G1‐G5 groups and quantitative analysis. C) Proportion of CD8+ T cells in tumor tissues from G1‐G5 groups and quantitative analysis. D) Proportion of cytotoxic T cells in tumor tissues from G1‐G5 groups and quantitative analysis. E,F) Double immunofluorescence staining of CD8a and CD86 expression in tumor tissues from G1 (PRL@RBCM‐cRGD) and G5 (siPFKFB4/PRL_PTX_@RBCM‐cRGD) groups, along with quantitative analysis (n = 3). G–J) Serum ELISA analysis of different cytokine levels in G1‐G5 groups. ^*^ *p* < 0.05, ^**^ *p* < 0.01, ^***^ *p* < 0.001.

Previous in vitro studies demonstrated the role of the PFKFB4‐targeted nanoparticle co‐delivery system in modulating the SCLC immune microenvironment. In vivo experiments further validated this phenomenon. As shown in Figure 8G, HMGB1 expression in the G5 group was 6.9 fold higher than in the G1 group, indicating significant immunogenic cell death (ICD) induction. The PFKFB4‐targeted nanoparticle co‐delivery system increased the proportion of M1 macrophages (up to 56.7%) while decreasing M2 macrophages (from 50.6% to 27.0%), resulting in an M1/M2 ratio of 1.92 (**Figure** **9**A; Figure S11, Supporting Information). The maturation of dendritic cells (DCs), a critical component of antigen presentation, also improved, with mature DCs accounting for 40.5% of total DCs (Figure 9B). CD8+ T cells, essential for antitumor immunity, increased to 32.0%, with a significant rise in GZMA+ CD8+ T cells (Figure 9C,D). Immunofluorescence further confirmed enhanced CD8α and CD86 expression in the G5 group compared to G1 (Figure 9E,F). Cytokine secretion, including IFN‐γ, IL‐2, IL‐12, and TNF‐α, was also markedly elevated in the G5 group (Figure 9G–J), collectively demonstrating robust immune activation.

**Figure 10:**
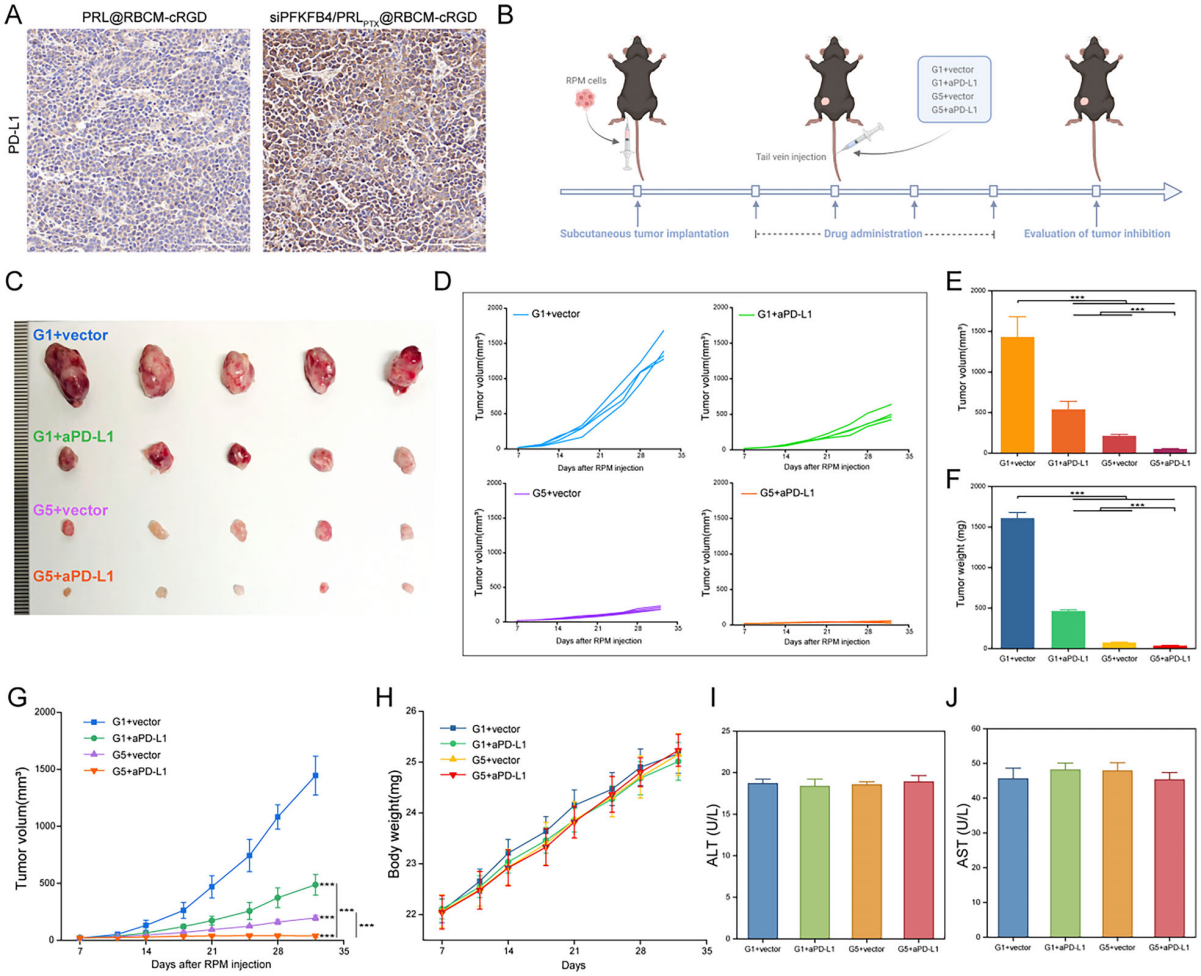
Combination Therapy of siPFKFB4/PRL_PTX_@RBCM‐cRGD and Anti‐PD‐L1 Antibody Inhibits SCLC. A) PD‐L1 expression levels in tumor tissues from tumor‐bearing mice treated with PRL@RBCM‐cRGD and siPFKFB4/PRL_PTX_@RBCM‐cRGD. B) Combination treatment with siPFKFB4/PRL_PTX_@RBCM‐cRGD and anti‐PD‐L1 antibody. C–G) Tumor volume and tumor weight differences in mice from different treatment groups (PRL@RBCM‐cRGD+vector, PRL@RBCM‐cRGD+anti‐PD‐L1, siPFKFB4/PRL_PTX_@RBCM‐cRGD+vector, siPFKFB4/PRL_PTX_@RBCM‐cRGD+anti‐PD‐L1, n = 5 per group). H) Body weight changes in mice during the combination therapy process (n = 5 per group). I,J) Liver function indicators in mice from different groups (n = 3).

Interestingly, PD‐L1 expression in G5 group tumor tissues was higher than in G1, prompting further exploration of combined therapy with PD‐L1 antibodies (**Figure** **10**A; Figure S12D, Supporting Information). As shown in Figure 10C–G, siPFKFB4/PRL_PTX_@RBCM‐cRGD, and PD‐L1 antibodies each achieved certain antitumor effects as monotherapies; however, their combination significantly enhanced tumor suppression. Notably, no significant changes in mouse body weight (Figure 10H) or liver and kidney function (Figure 10I,J; Figure S12A—C, Supporting Information) were observed, underscoring the safety of this combined approach. Together, these results highlight the synergistic potential of the biomimetic codelivery system with PD‐L1 antibodies in enhancing antitumor efficacy.

## 3 Discussion

As one of the drug options following first‐line resistance in SCLC patients, paclitaxel has not become the preferred choice for second‐line treatment. The main reasons are its suboptimal efficacy in SCLC treatment and the hypersensitivity and toxicity associated with conventional paclitaxel due to the use of solvents. To enhance the efficacy of paclitaxel and reduce solvent‐induced hypersensitivity reactions, novel drug delivery technologies such as liposomes, microspheres, and nanoparticles have been proposed for their application in SCLC treatment.^[^ ^7^ ^]^ Additionally, exploring new targets to increase paclitaxel sensitivity in SCLC is another approach to enhancing its therapeutic efficacy. As shown in the results in Figure 1, SCLC patients with high PFKFB4 expression have a poorer prognosis, suggesting that PFKFB4 plays a critical role in the development and progression of SCLC. Single‐cell data analysis and immune cell infiltration analysis indicate that PFKFB4 is predominantly expressed in macrophages within the immune microenvironment of SCLC. Furthermore, the expression level of PFKFB4 is negatively correlated with CD8+ T cells, macrophages, dendritic cells, and other cell types integral to antitumor immunity. This suggests that PFKFB4 may regulate the immune microenvironment of SCLC and primarily contribute to the formation of an immunosuppressive microenvironment. These findings indicate that PFKFB4 has potential as a novel therapeutic target for SCLC. Moreover, the knockdown of PFKFB4 in two SCLC cell lines significantly reduced the IC50 of paclitaxel, demonstrating that PFKFB4 knockdown markedly enhances the sensitivity of SCLC to paclitaxel. Targeting or inhibiting PFKFB4 in combination with paclitaxel treatment may yield improved antitumor effects in SCLC.

In this study, a pH‐sensitive cationic liposome was employed as a nanocarrier to co‐deliver PTX and siPFKFB4, with the outermost layer coated with red blood cell membrane vesicles conjugated with cRGD. The nanoparticles had an average diameter of 176.60 nm, and their successful encapsulation was confirmed by zeta potential measurements and electron microscopy images (Figure 2B,C). Red blood cell membrane vesicles, serving as a natural coating, are widely used in drug delivery studies due to their inherent biocompatibility, enhanced material stability, and ability to evade immune clearance.^[^ ^21^, ^22^, ^23^ ^]^ Consistent with previous studies, the retention of proteins on the red blood cell membranes, the expression of CD47, and uptake experiments using RAW264.7 cells suggest that the encapsulation of the codelivery system by red blood cell membranes enables the nanoparticles to evade macrophage phagocytosis and achieve prolonged circulation in vivo. Cytotoxicity assays, hematoxylin and eosin (H&E) staining of various organs, and blood biochemical analysis demonstrate that the nanoparticle co‐delivery system exhibits high biosafety both in vitro and in vivo. Conventional nanodrugs primarily rely on the enhanced permeability and retention (EPR) effect for passive tumor accumulation.^[^ ^24^, ^25^ ^]^ To enable active tumor targeting, cRGD, a commonly used targeting peptide for nanomaterial modification,^[^ ^26^, ^27^ ^]^ was conjugated to the red blood cell membrane vesicles. Results from Figure 2K,L, and 7A,B show that RBCM‐cRGD improves tumor accumulation of the nanoparticles and enhances drug uptake by tumor cells. Collectively, these findings indicate that the biomimetic codelivery system exhibits excellent safety and high tumor‐targeting capability, showing promise for future clinical applications in SCLC treatment.

Notably, nanoparticle‐mediated targeting of PFKFB4 demonstrated potent antitumor efficacy in both in vitro and in vivo experiments. Based on the aforementioned results, the enhanced tumor‐killing effect of the system compared to PTX or PFKFB4 inhibition alone can be attributed to the increased sensitivity of SCLC to PTX upon PFKFB4 downregulation. Ferroptosis, a non‐apoptotic form of cell death driven by iron‐dependent lipid peroxidation, is often accompanied by aberrant expression of iron metabolism‐related molecules such as GPX4^[^ ^28^, ^29^ ^]^ and xCT.^[^ ^30^ ^]^ Chemoresistant tumor cells frequently exhibit high sensitivity to ferroptosis, suggesting its potential to enhance chemotherapy sensitivity and reverse drug resistance.^[^ ^31^ ^]^ Inducing ferroptosis has been reported to improve PTX sensitivity in several cancer types, including triple‐negative breast cancer,^[^ ^32^, ^33^ ^]^ esophageal cancer,^[^ ^34^ ^]^ and hepatocellular carcinoma.^[^ ^35^ ^]^ Similarly, our electron microscopy findings revealed significant ferroptotic features in SCLC cells treated with the nanoparticle co‐delivery system. Ferroptosis‐related proteins such as GPX4 and xCT were downregulated, whereas ACSL4 expression was markedly upregulated. Treatment also resulted in increased intracellular levels of MDA and ROS, along with iron ion accumulation (Figure 4E,F). Elevated ROS levels and decreased GPX4 expression were further validated in tumor tissues from treated mice (Figure 8F,G). Furthermore, in both the CCK‐8 and colony formation assays, we observed that the ferroptosis inhibitor Fer‐1 partially mitigates the inhibitory effect of the co‐delivery system on the proliferation of SCLC cells (Figure 4I,J). These results collectively indicate that the targeting PFKFB4 nanoparticle co‐delivery system induces ferroptosis in SCLC cells by suppressing PFKFB4, thereby enhancing the antitumor efficacy of PTX.

It is well established that the limited clinical benefit of ICIs in SCLC patients is largely attributable to the unique immunosuppressive tumor microenvironment characteristic of this malignancy. The immune microenvironment of SCLC tumors is generally acidic, enriched with glutathione and hydrogen peroxide,^[^ ^36^ ^]^ in addition to sharing key pathophysiological features common to solid tumors such as hypoxia.^[^ ^37^ ^]^ Moreover, SCLC exhibits low levels of tumor‐infiltrating lymphocytes,^[^ ^38^ ^]^ significantly reduced expression of programmed PD‐L1,^[^ ^39^, ^40^ ^]^ deficient expression of major histocompatibility complex class I (MHC‐I) molecules,^[^ ^41^ ^]^ and absent expression of MHC class II proteins,^[^ ^42^ ^]^ all of which contribute to its classification as a cold TIME. These factors pose substantial challenges to the efficacy of ICIs in SCLC treatment. Consequently, there is a pressing need to identify novel therapeutic targets or develop advanced drug delivery systems to remodel the SCLC immune microenvironment and enable more patients to benefit from immunotherapy.

Recent evidence suggests that inducing ferroptosis not only inhibits tumor growth but also activates the immune system and enhances immunotherapy responses.^[^ ^43^, ^44^ ^]^ During ferroptosis, the tumor immune microenvironment may shift, suppressing tumor progression and metastasis.^[^ ^45^ ^]^ Furthermore, ferroptosis‐induced ICD exhibits potent immunostimulatory effects through the release of DAMPs, such as HMGB1, which activate dendritic cell maturation and enhance cytotoxic T lymphocyte infiltration, thereby bridging oxidative lipid peroxidation with antitumor immunity.^[^ ^46^ ^]^ Our results demonstrate that the elevated HMGB1 expression observed in mouse tumor tissues following treatment with engineered lipid nanoparticles indicates that immunogenic cell death occurred in SCLC cells. Furthermore, in vitro co‐culture experiments simulating the SCLC immune microenvironment, combined with in vivo studies, revealed significant immune modulation. Focused analysis of tumor‐associated macrophage (TAM) polarization, dendritic cell (DC) maturation, and cytotoxic T‐cell infiltration showed that the biomimetic codelivery system shifted TAMs toward an M1 phenotype, as evidenced by increased CD86 and MHC‐II expression and decreased CD206 and Arg1 expression. Additionally, cytokines such as TNF‐α were significantly elevated, indicating a pro‐inflammatory state. These findings were corroborated in vivo, with CD80/CD86 double‐positive DCs accounting for 30–40% of the population. Furthermore, cytotoxic T‐cell infiltration was significantly increased in tumor tissues from treated mice, indicating an activated immune microenvironment. These results suggest that the co‐delivery system amplifies ferroptosis‐induced ICD, thereby activating the microenvironment of SCLC.

Transcriptomic sequencing and enrichment analyses revealed the activation of cytokine‐related pathways, particularly the JAK‐STAT pathway, which is known to regulate immune responses in the tumor microenvironment.^[^ ^47^, ^48^ ^]^ Western blot analysis confirmed JAK‐STAT pathway activation in DCs and macrophages, underscoring its critical role in reversing the immunosuppressive microenvironment of SCLC. Notably, PD‐L1 expression was upregulated in tumor tissues following treatment with PFKFB4‐targeted nanoparticles. Previous studies have shown that increased PD‐L1 expression enhances the efficacy of anti‐PD‐L1 antibodies.^[^ ^49^, ^50^ ^]^ Combining siPFKFB4/PRL_PTX_@RBCM‐cRGD with anti‐PD‐L1 antibodies resulted in synergistic therapeutic effects, significantly suppressing tumor growth, thereby providing a strong theoretical basis for combining chemotherapy with immunotherapy in clinical applications.

While the biomimetic codelivery system demonstrated significant antitumor efficacy and remarkable modulation of the tumor immune microenvironment in SCLC, this study has certain limitations. Variability in product batches and the complexity of the tumor immune microenvironment pose challenges for its clinical translation. The immunomodulatory effects and potential biological interactions of positively charged nanoparticles in vivo warrant comprehensive investigation. Additionally, the co‐delivery system was not evaluated in multiple SCLC subtypes or patient‐derived xenograft (PDX) models. Further research is needed to elucidate precise regulatory mechanisms and address these challenges to facilitate clinical applications.

## 4 Conclusion

This study proposes a biomimetic red blood cell membrane‐based co‐delivery system for PTX and siPFKFB4. This system demonstrates excellent biocompatibility and prolonged circulation in vivo, with the capability of actively targeting tumors and responding to the acidic tumor microenvironment. It enhances therapeutic sensitivity by promoting ferroptosis in SCLC cells. Furthermore, the PFKFB4‐targeted nanoparticle co‐delivery system induces immunogenic cell death in tumor cells, leading to the secretion of relevant cytokines and activation of the JAK‐STAT signaling pathway in DCs and tumor‐associated macrophages (TAMs). This results in the maturation of DCs, polarization of TAMs toward the M1 phenotype, and activation of effector T cells, thereby enhancing antitumor immunity. The combination of the biomimetic codelivery system with anti‐PD‐L1 antibodies synergistically improves the efficacy of antitumor immunotherapy.

## 5 Experimental Section

### Cell Culture

Human SCLC cell lines NCI‐H446 and DMS114, along with RAW264.7 murine macrophages, were purchased from Procell (Wuhan, China). The murine SCLC cell line RPM was derived from primary orthotopic lung tumors of Rb1^flox/flox^, Trp53^flox/flox^, and Myc^LSL/LSL^ (RPM) mice, obtained from Jackson Laboratory (#02 9971). NCI‐H446 and RPM cells were cultured in 1640 medium supplemented with 10% (v/v) fetal bovine serum (FBS) under a 5% CO₂ atmosphere at 37 °C. DMS114 and RAW264.7 cells were cultured in DMEM medium supplemented with 10% FBS under the same conditions.

### Reagents and Materials

Reagents included fetal bovine serum (Procell, Wuhan, China), DMEM and 1640 media (Nanjing KeyGen Biotech Inc., China), ROS probes (MCE, USA), MDA assay kits (Solarbio, Beijing, China), paclitaxel (Rhawn, Guangzhou, China), Cell Counting Kit‐8 (GLPBIO, USA), and Cell Ferrous Iron Colorimetric Assay Kit (Elabscience, Wuhan, China). Edu assay kits and Live/Dead cell double‐staining kits were purchased from Abbkine Scientific Co., Ltd (Wuhan, China). Lipids used in nanoparticle preparation included 1,2‐dipalmitoyl‐sn‐glycero‐3‐phosphorylcholine (DPPC), cholesterol, 1,2‐dioleoyl‐3‐trimethylammonium propane (DOTAP), DSPE‐PEG2K‐cRGD, and 1,2‐distearoyl‐sn‐glycero‐3‐phosphoethanolamine‐poly(2‐ethyl‐2‐oxazoline) (DSPE‐PEOz).

### Synthesis of siPFKFB4/PRL_PTX_@RBCM‐cRGD

Cationic liposomes were synthesized using a thin‐film dispersion method. Appropriate amounts of DPPC, cholesterol, DOTAP, DSPE‐PEOz, Cy5.5, and PTX were dissolved in a chloroform‐methanol mixture. The solution was ultrasonicated for uniform mixing and transferred to a rotary evaporator, where it was rotated at 40 °C for 15 min to form a lipid film. The film was hydrated with DEPC‐treated water, ultrasonicated, and extruded repeatedly through 200 nm polycarbonate membranes using a liposome extruder (Avanti, USA) to obtain PRL_PTX_. PRL_PTX_ and siPFKFB4 were mixed in an enzyme‐free EP tube at a mass ratio determined by gel retardation assay, ultrasonicated, and incubated at room temperature for 15–20 min to allow binding, yielding siPFKFB4/PRL_PTX_, which was stored at 4 °C.

Mouse blood was obtained via cardiac puncture and centrifuged at 3000 rpm at 4 °C for 20 min to remove serum. The red blood cells (RBCs) were washed with PBS, centrifuged, and resuspended in 0.25 × PBS at a 1:40 volume ratio. The mixture was incubated at 4 °C for 2 h to lyse the RBCs, followed by high‐speed centrifugation at 12000 rpm at 4 °C for 20 min. The lysed RBCs were washed with 0.25 × PBS hypotonic solution and centrifuged to obtain pale red or white RBC membranes. The membranes were extruded sequentially through 800, 400, and 200 nm polycarbonate membranes to produce red blood cell membrane vesicles (RBCM). RBCM was mixed with PBS‐dissolved cRGD and ultrasonicated, followed by incubation at 37 °C for 20 min to obtain RBCM‐cRGD. Finally, siPFKFB4/PRL_PTX_ and RBCM‐cRGD were mixed in an appropriate ratio and ultrasonicated at 100 W and 59 Hz for 5 min. The mixture was extruded through a liposome extruder, sequentially passing through 800, 400, and 200 nm polycarbonate membranes to obtain siPFKFB4/PRL_PTX_@RBCM‐cRGD with uniform particle size.

### Gel Retardation Assay

A 2% agarose gel was prepared by dissolving 0.4 g of agarose in 20 mL of 0.5×TBE buffer with gentle shaking. The mixture was microwaved until the agarose fully dissolved. After cooling to ≈60 °C, 2 µL of Gel Red (Biosharp, China) was added and gently mixed before pouring into a gel mold. The gel was left to solidify. Samples from different groups were loaded into separate wells of the gel. Electrophoresis was performed at 100 V for 20 min. The gel was imaged to assess sample migration.

### SDS‐PAGE Electrophoresis and Western Blot

For protein extraction, samples were lysed with RIPA buffer containing 1% protease and phosphatase inhibitors. After 30 min on ice, the lysates were centrifuged, and the supernatant containing extracted proteins was collected. Protein concentrations were quantified using the BSA method. Equal amounts of protein were loaded onto pre‐prepared 10% SDS‐PAGE gels for electrophoresis. SDS‐PAGE Electrophoresis: The gels were stained with Coomassie Brilliant Blue for 30 min and destained until clear protein bands were visible against a transparent background. Bands were visualized using a gel imaging system. Western Blot: Proteins were transferred to a polyvinylidene fluoride (PVDF) membrane, which was then blocked with skim milk for 120 min. After washing, the membrane was incubated with primary antibodies at 4 °C for 16–18 h. Secondary antibodies were incubated at room temperature for ≈120 min. Enhanced chemiluminescence (ECL) reagents were used to detect protein bands, which were visualized using a chemiluminescence imaging system. Details of antibodies used are provided in the Supporting Information.

### Cell Viability Assay

Cells were seeded at a density of 6 × 10^3^ per well in 96‐well plates. After adhesion, cells were treated with PTX or nanoparticles at different concentrations and incubated for 24 h. A complete medium containing 10% CCK‐8 solution was added to each well and incubated for an additional 2 h. Absorbance was measured using a microplate reader. Each experiment was independently repeated three times.

### Cellular Uptake Assay

Cells were seeded onto confocal dishes. After adhesion, 500 µL of complete medium containing siNC/PRL or siNC/PRL@RBCM‐cRGD was added. After incubation in the dark for 6, 12, and 24 h, the dishes were retrieved, and cells were fixed with 4% paraformaldehyde for 20 min. Nuclei were stained with DAPI, and coverslips were mounted. Cellular uptake was visualized using a confocal microscope.

### Colony Formation Assay

Cells were seeded into six‐well plates and allowed to adhere. The cells were then treated with media containing nanoparticles or drugs and incubated for 24 h. Fresh medium was replaced every 2–3 days. Once visible colonies formed, cells were fixed with paraformaldehyde for 20 min, washed with PBS, and stained with crystal violet. The stained plates were washed with PBS, air‐dried, and photographed.

### Live/Dead Staining Assay

Cells were seeded in confocal dishes and incubated in a CO₂ incubator at 37 °C for 24 h. Subsequently, 500 µL of medium containing nanoparticles or drugs were added, and cells were incubated for another 24 h. After two PBS washes, 0.5 mL of staining solution was added, and the cells were incubated in the dark at 37 °C for 30 min. After another PBS wash, coverslips were mounted, and fluorescence was immediately observed under a fluorescence microscope.

### EdU Assay

Reagents from the EdU assay kit were prepared in advance. Cells were seeded into confocal dishes and treated with nanoparticles or drugs for 24 h. A 2× EdU solution, prewarmed to 37 °C, was diluted to 1× final concentration in the medium and added to the cells. After 2 h of incubation, cells were fixed, washed, and permeabilized. A 100 µL Click‐iT reaction mixture was added to each sample, and cells were incubated at room temperature in the dark for 30 min. After washing with 1× BSA wash solution for 5 min, nuclei were stained, coverslips were mounted, and fluorescence was observed under a fluorescence microscope.

### ROS Detection

The ROS detection probe H_2_DCFDA was dissolved in DMSO to prepare a 10 mM stock solution, which was further diluted before use. Cells were incubated with PBS containing 5 µM H_2_DCFDA at 37 °C in the dark for 30 min. After washing with PBS, fluorescence was observed using a confocal microscope.

### MDA Detection

For each sample, 1 mL of MDA extraction solution was added to 5 million cells, followed by ultrasonic lysis. The supernatant was collected and kept on ice. A working solution was prepared by mixing 300 µL of MDA reagent, 100 µL of the sample, and 100 µL of reagent three. The mixture was incubated in a 100 °C water bath for 60 min, cooled on ice, and centrifuged to collect the supernatant. Absorbance was measured at 532 and 600 nm using a microplate reader. The MDA content for each group was calculated based on the absorbance and cell count.

### Iron Ion Detection

Cell samples were collected, and 0.2 mL of reagent one was added 1 × 10^6^ per cell. After lysis and centrifugation, the supernatant was collected and kept on ice. For the assay, 80 µL of standard solution was added to the standard wells of a microplate, and 80 µL of the sample was added to the test and control wells. Then, 80 µL of reagent two was added to the control wells, while 80 µL of reagent three was added to the test and standard wells. The solutions were mixed and incubated at 37 °C for 10 min. OD values at 593 nm were measured using a microplate reader, and the iron ion content in the samples was calculated based on the standard curve.

### BMDM and BMDC Extraction and Induction

Bone marrow was harvested from the femurs and tibias of 6‐week‐old C57BL/6 mice using pre‐chilled PBS. The marrow was filtered and dissociated to ensure single‐cell suspensions. Cells were centrifuged, and the supernatant was discarded. Red blood cell lysis buffer was added to the cell pellet for 3 min, followed by the addition of PBS to stop lysis. After further centrifugation and washing steps, the cells were transferred to culture plates. BMDC Induction: Cells were cultured in RPMI‐1640 medium supplemented with 20 ng mL^−1^ GM‐CSF and 10 ng mL^−1^ IL‐4 for 6 days. BMDM Induction: Cells were cultured in DMEM supplemented with 20 ng mL^−1^ M‐CSF for 6 days. Induced BMDCs and BMDMs were used for subsequent experiments.

### SCLC Syngeneic Tumor Model

C57BL/6 male mice (6–8 weeks old) were subcutaneously injected with 100 µL of PBS containing 1 × 10^6^ RPM cells into the right inguinal region. After 7 days, mice were randomly divided into five groups: PRL@RBCM‐cRGD, PTX, siNC/PRL@RBCM‐cRGD, siPFKFB4/PRL@RBCM‐cRGD, and siPFKFB4/PRL_PTX_@RBCM‐cRGD. PTX was administered at a dose of 10 mg kg^−1^ once a week for four weeks. Tumor volume and mouse weight were recorded every three days. Tumor volume was calculated using the formula: *Volume = 0.5 × Length × Width^2^*. At the end of the experiment, blood was collected via cardiac puncture, and serum was extracted. Mice were euthanized using CO₂, and tissues including the heart, liver, spleen, lungs, kidneys, and tumors were harvested for subsequent analysis. Combination Therapy with aPD‐L1: Tumor‐bearing mice were randomized into four groups: PRL@RBCM‐cRGD + vector, PRL@RBCM‐cRGD + aPD‐L1, siPFKFB4/PRL_PTX_@RBCM‐cRGD + vector, and siPFKFB4/PRL_PTX_@RBCM‐cRGD + aPD‐L1. All animal studies were approved by the Ethics Committee of Zhujiang Hospital, Southern Medical University (Approval Number: LAEC‐2023‐223) and conducted in compliance with the principles of the Helsinki Declaration.

### In Vivo Uptake Study

C57BL/6 mice bearing subcutaneous RPM cell tumors in the right inguinal region were randomly divided into two groups. Each group received tail vein injections of PRL or PRL@RBCM‐cRGD. Mice were imaged at 2, 6, and 24 h post‐injection using an IVIS Spectrum imaging system. At 24 h, mice were euthanized, and organs including the heart, liver, spleen, lungs, kidneys, and tumors were harvested to evaluate biodistribution. Data analysis was performed using the Living Image software.

### Immunohistochemistry and immunofluorescence

Tumor tissues from different treatment groups were dehydrated, embedded, and sectioned to obtain paraffin slices. Sections were deparaffinized, subjected to antigen retrieval, and blocked. Primary antibodies were added, and sections were incubated overnight at 4 °C. The next day, sections were washed with PBS and incubated with secondary antibodies at room temperature in the dark for 1 h. DAB or TAB reagent was added for further incubation, followed by DAPI staining for nuclei visualization. Slides were sealed and imaged under a fluorescence microscope. This study was approved by the Ethics Committee of Zhujiang Hospital (2024‐KY‐129‐01), Southern Medical University, and conducted in compliance with the Declaration of Helsinki.

### Flow Cytometry

Tumor samples were digested and processed to single‐cell suspensions, followed by red blood cell lysis. Cells were incubated with surface antibodies in the dark for 30 min, fixed, permeabilized, and stained with intracellular antibodies. The following markers were used: M1 Macrophages: CD45⁺F4/80⁺CD86⁺, M2 Macrophages: CD45⁺F4/80⁺CD206⁺, DCs: CD45⁺CD11c⁺MHC‐II⁺, CD8⁺ T Cells: CD45⁺CD3⁺CD8⁺. The samples were analyzed using a CytoFLEX flow cytometer. Data were processed and visualized using FlowJo 10.8.1 software. Detailed information about the antibodies used is provided in the Supporting Information.

### Data Analysis

All data were obtained from three independent experiments and expressed as mean ± standard error of the mean (SEM). Statistical analyses were performed using SPSS software. The following tests were employed: Independent Samples *t*‐Test, Wilcoxon Test, and One‐Way ANOVA, Differences were considered statistically significant if *p* < 0.05. Graphical representations were created using Origin 2021 software, and final figures were arranged using Adobe Illustrator 2021.

## Conflict of Interest

The authors declare no conflict of interest.

## Author Contributions

X.L., J.H., and H.Y. contributed equally to this work. This study was a collaborative effort of all authors. X.L. was responsible for the synthesis and characterization of nanoparticles, experimental validation, data analysis, and manuscript preparation. J.H. conducted the animal experiments. H.Y. performed sequencing data analysis. P.L. and W.Z. contributed to data analysis and verification. C.C. was primarily involved in the cell experiments. C.Z. managed the primary cell culture and induction. T.W. oversaw the overall project. B.T. was in charge of project management, experimental design, and manuscript review. J.Z. supervised the overall progress of the project and facilitated the acquisition of funding from the National Natural Science Foundation of China. All authors approved the final version of the manuscript for submission.

## Supporting information



Supporting Information

## Data Availability

The data that support the findings of this study are available in the supplementary material of this article.
