# Supplementary material for: Targeting PFKFB4 Biomimetic Codelivery System Synergistically Enhances Ferroptosis to Suppress Small Cell Lung Cancer and Augments the Efficacy of Anti‐PD‐L1 Immunotherapy
Source: Adv Sci (Weinh). 2025 Apr 11;12(22):2417374. doi: 10.1002/advs.202417374 (PMC12165097; doi:10.1002/advs.202417374)
Supplement: Supplementary file 1 — Supporting Information [file ADVS-12-2417374-s001.docx]

### Supporting Information

**Authors' Information**

**Xiang Liu**, Department of Oncology, Zhujiang Hospital, Southern Medical University, Guangzhou, Guangdong Province 510282, China, Email: lxczzx1116@163.com.

**Jingjun He**, Department of Oncology, Zhujiang Hospital, Southern Medical University, Guangzhou, Guangdong Province 510282, China, Email: 823351077@qq.com.

**Haoxuan Ying**, Department of Oncology, Zhujiang Hospital, Southern Medical University, Guangzhou, Guangdong Province 510282, China, Email: nfykdxyinghx@163.com.

**Cuiying Chen**, Department of Oncology, Zhujiang Hospital, Southern Medical University, Guangzhou, Guangdong Province 510282, China, Email: chency1701@163.com.

**Chongyang Zheng**, Department of Oncology, Zhujiang Hospital, Southern Medical University, Guangzhou, Guangdong Province 510282, China, Email: zhengchongyang2022@163.com.

**Peng Luo**, Department of Oncology, Zhujiang Hospital, Southern Medical University, Guangzhou, Guangdong Province 510282, China, Email: luopeng@smu.edu.cn.

**Weiliang Zhu**, Department of Oncology, Zhujiang Hospital, Southern Medical University, Guangzhou, Guangdong Province 510282, China, Email: duarion@126.com.

**Ting Wei**, Department of Oncology, Zhujiang Hospital, Southern Medical University, Guangzhou, Guangdong Province 510282, China, Email: weitingyouyou@qq.com.

**Bufu Tang**, Department of Radiation Oncology, Zhongshan Hospital Affiliated to Fudan University, Shanghai 200032, China, Email: tangbufu@zju.edu.cn.

**Jian Zhang**, Department of Oncology, Zhujiang Hospital, Southern Medical University, Guangzhou, Guangdong Province 510282, China, Email: zhangjian@i.smu.edu.cn.

**Supplementary Tables**

Table S1. The target sequence of siRNA

| RNAi | Sense sequence (5’ to 3’) | Antisense sequence (5’ to 3’) |
| --- | --- | --- |
| siRNA-NC | UUCUCCGAACGUGUCACGUTT | / |
| siPFKFB4-1 | CCAACUGCCCAACUCUCAUTT | / |
| siPFKFB4-2 | GGACUUCAUGAGGCGCAUUTT | / |

Table S2. Antibodies used in the manuscript

| Name | Species | Dilution | Company | Cat number |
| --- | --- | --- | --- | --- |
| PFKFB4 | Rabbit | WB: 1:1000  IHC: 1:500 | Abcam | ab137785 |
| CD47 | Mouse | WB: 1:200 | Santa Cruz | sc-12730 |
| PCNA | Rabbit | IF: 1:500 | Proteintech | 10205-2-AP |
| xCT | Rabbit | WB: 1:1000  IF: 1:400 | Abcam | ab307601 |
| ACSL4 | Rabbit | WB: 1:1000 | Proteintech | 22401-1-AP |
| GPX4 | Mouse | IF: 1:500 | Proteintech | 67763-1-Ig |
| HSP90 | Mouse | WB: 1:5000 | Proteintech | 13171-1-AP |
| GAPDH | Rabbit | WB: 1:5000 | Proteintech | 10494-1-AP |
| CD86 | Rabbit | IF: 1:500 | Proteintech | 13395-1-AP |
| Arg1 | Mouse | IF: 1:500 | Proteintech | 66129-1-Ig |
| P-JAK1 | Rabbit | WB: 1:1000 | Affinity | AF2012 |
| JAK1 | Rabbit | WB: 1:1000 | Affinity | AF5012 |
| P-STAT1 | Rabbit | WB: 1:1000 | Affinity | AF3300 |
| STAT1 | Rabbit | WB: 1:1000 | Affinity | AF6300 |
| β-Actin | Mouse | WB: 1:4000 | Abcam | ab8226 |
| Ki67 | Rabbit | IHC: 1:4000 | Proteintech | 28074-1-AP |
| HMGB1 | Rabbit | IF: 1:200 | CST | 6893 |
| CD8a | Mouse | IF: 1:200 | Abcam | ab217344 |
| PD-L1 | Rabbit | IHC: 1:200 | Cell Signaling  Technology | #13684 |
| Goat anti Rabbit  IgG-HRP | Goat | IF: 1:500 | Abcam | ab150077 |
| Goat anti Mouse  IgG-HRP | Goat | IF: 1:500 | Abcam | ab150118 |

Table S3. FACS antibodies

| Name | Fluorophore | Clone | Company | Cat number |
| --- | --- | --- | --- | --- |
| CD45 | FITC | 30-F11 | BioLegend | 103107 |
| CD8a | BV421 | 53-6.7 | BioLegend | 100753 |
| CD3 | AF700 | 17A2 | BioLegend | 100215 |
| CD11b | AF700 | M1/70 | BioLegend | 101222 |
| F4/80 | BV421 | BM8 | BioLegend | 123137 |
| CD86 | BV510 | GL-1 | BioLegend | 105039 |
| CD11c | AF700 | N418 | BioLegend | 117319 |
| I-A/I-E | BV421 | M5/114.15.2 | BioLegend | 107632 |
| CD80 | APC | 16-10A1 | BioLegend | 104713 |
| CD206 | APC | Y17-505 | Biosciences | 568808 |
| GZMA | APC | GzA-3G8.5 | eBioscience | 17-5831-82 |

**Supplementary Figures**


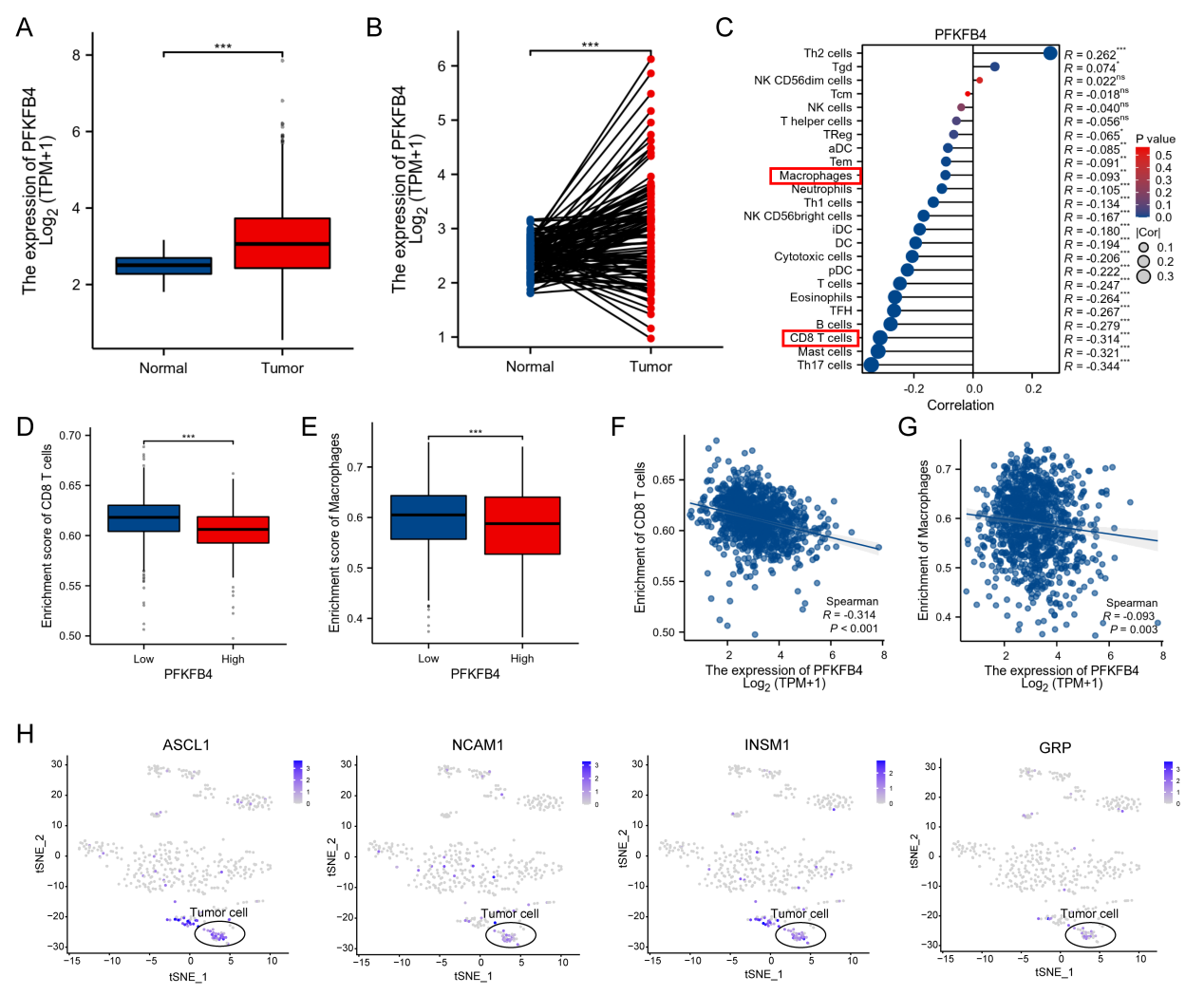


**Figure S1. Expression Levels of PFKFB4 in Lung Cancer and Immune Infiltration Analysis.** (A, B) Expression levels of PFKFB4 in normal tissues and lung cancer from the TCGA database. (A: unpaired samples, B: paired samples). (C) Correlation analysis between PFKFB4 expression and 24 types of immune cells. (D-F) Relationship between PFKFB4 expression levels and the infiltration of CD8+ T cells and macrophages. (H) Classify tumor cell populations using the four established diagnostic markers of neuroendocrine carcinoma (NEC): ASCL1, NCAM1, INSM1, and GRP.


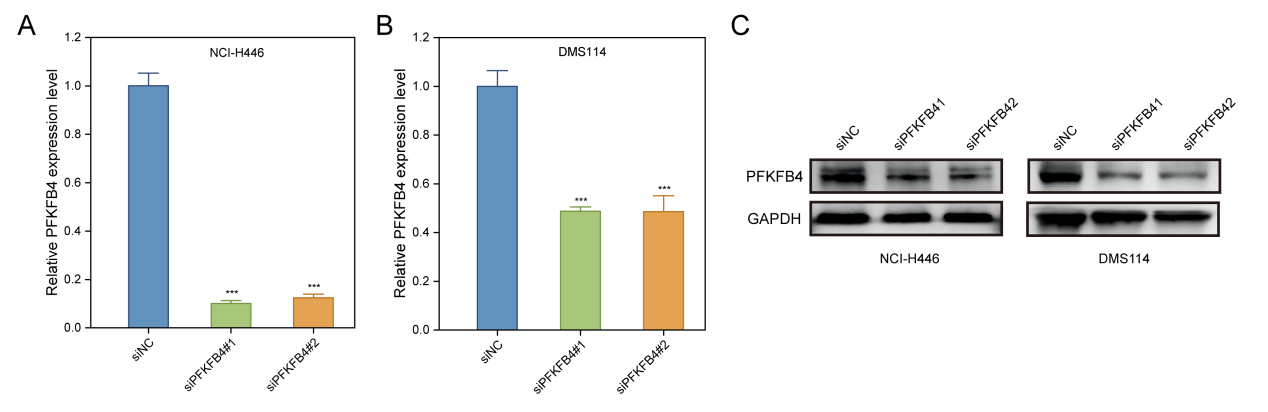


**Figure S2. Knockdown of PFKFB4 in NCI-H446 and DMS114 SCLC Cells using siRNA.** (A, B) RT-qPCR validation of the knockdown efficiency of two siRNA sequences in NCI-H446 and DMS114 cells. **P* < 0.05, ***P* < 0.01, ****P* < 0.001. (C) Western blot analysis of PFKFB4 protein expression in both cell lines after treatment with siNC, siPFKFB4#1, and siPFKFB4#2.


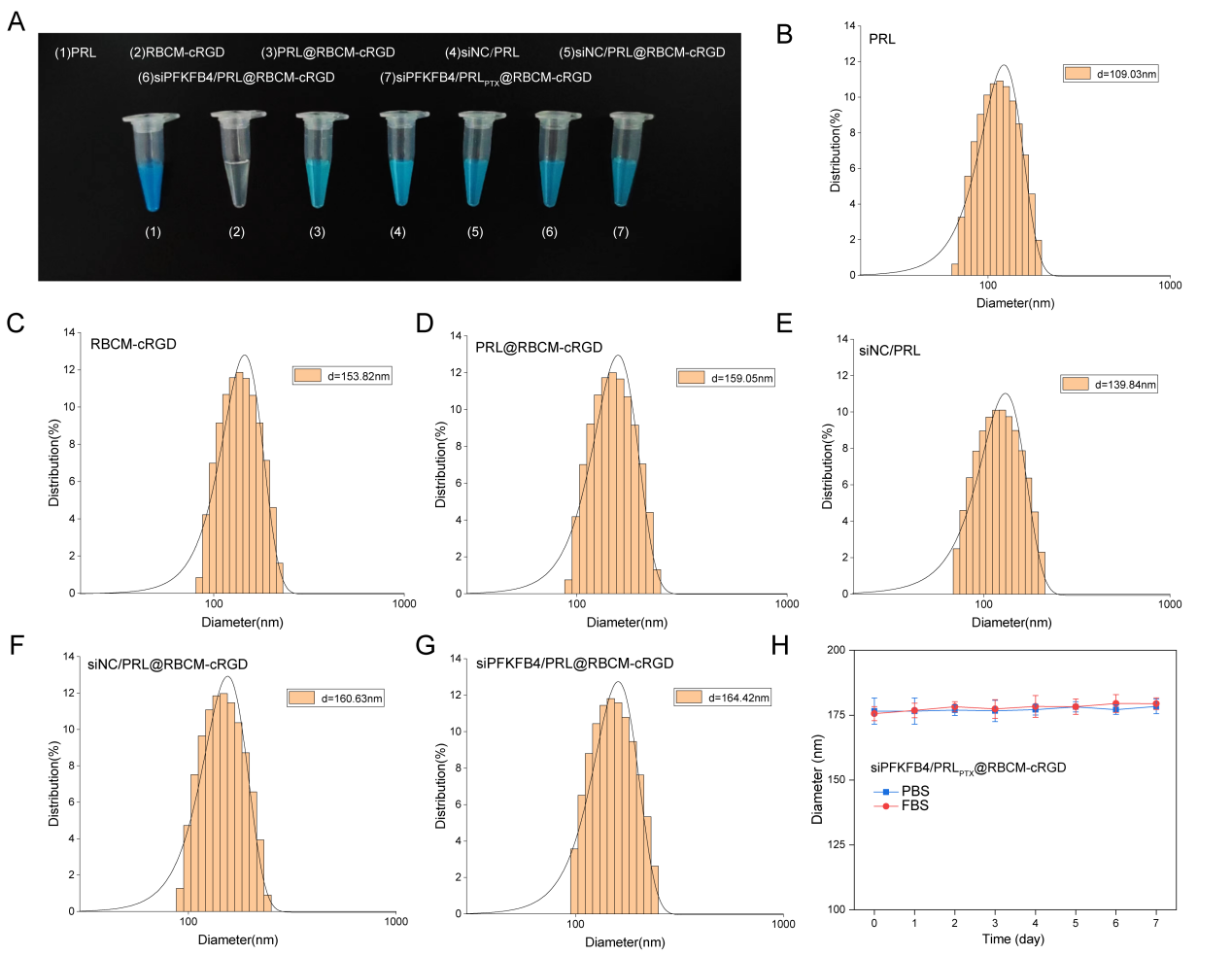


**Figure S3. Schematic Diagram and Particle Size of Different Nanoparticles.** (A) Schematic diagram of different Nanoparticles. (1) PRL, (2) RBCM-cRGD, (3) PRL@RBCM-cRGD, (4) siNC/PRL, (5) siNC/PRL@RBCM-cRGD, (6) siPFKFB4/PRL@RBCM-cRGD, (7) siPFKFB4/PRL_PTX_@RBCM-cRGD. (B-G) Particle size of different Nanoparticles. (H) Particle size variation of siPFKFB4/PRLPTX@RBCM-cRGD in FBS and PBS.


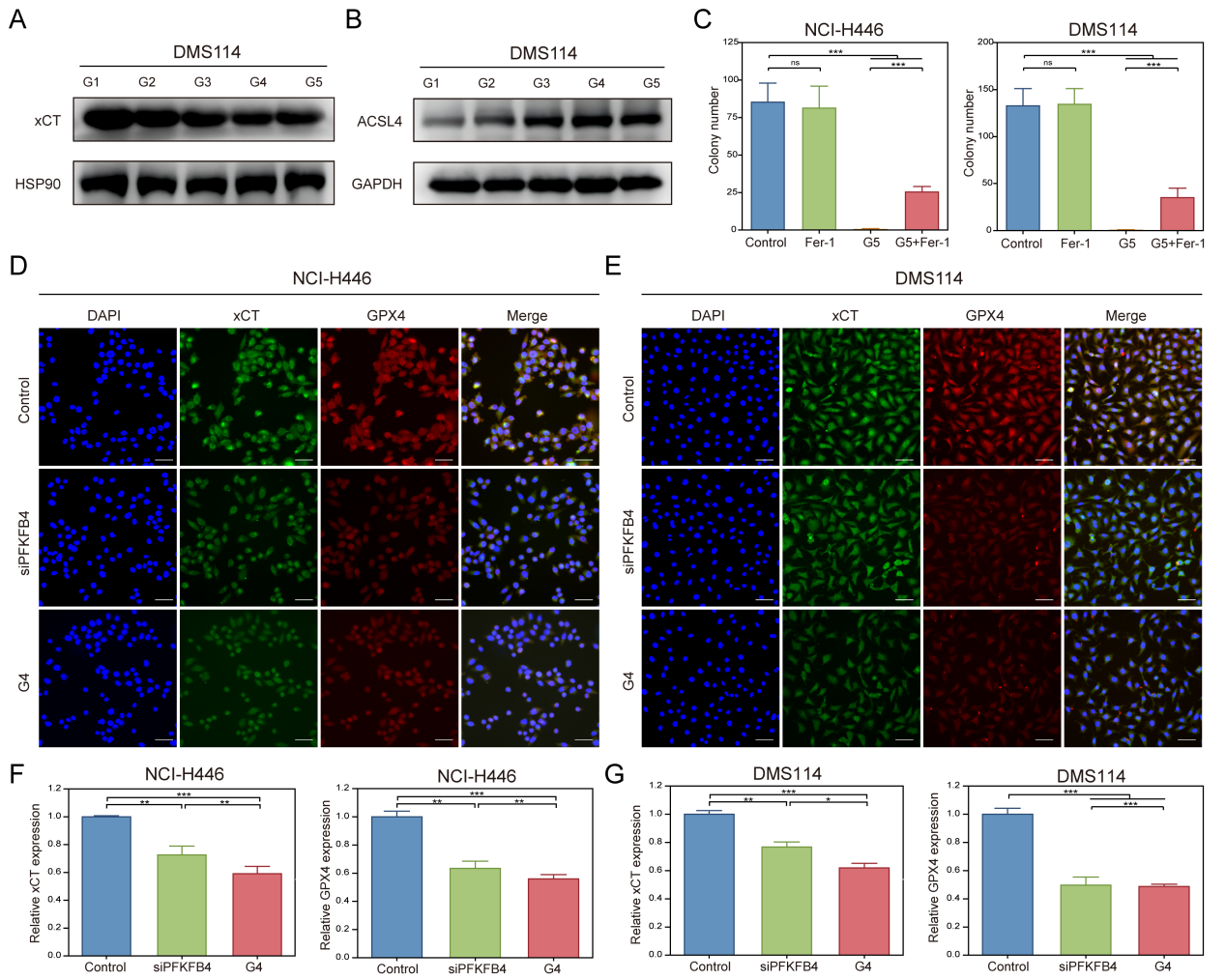


**Figure S4. The co-delivery system amplifies ferroptosis-mediated killing of SCLC cells.** (A, B)Expression Levels of xCT and ACSL4 Proteins in DMS114 Cells from G1-G5 Groups. (C) Colony formation assay comparing the proliferative capacity of SCLC cells across four groups: Control, Fer-1(ferroptosis inhibitor), G5, and G5 combined with Fer-1. (D, E) Immunofluorescence staining showing GPX4 and xCT expression levels in two cell lines treated with Control, siPFKFB4, and G4. (F, G) Quantitative analysis of GPX4 and XCT expression levels based on immunofluorescence staining. **P* < 0.05, ***P* < 0.01, ****P* < 0.001.


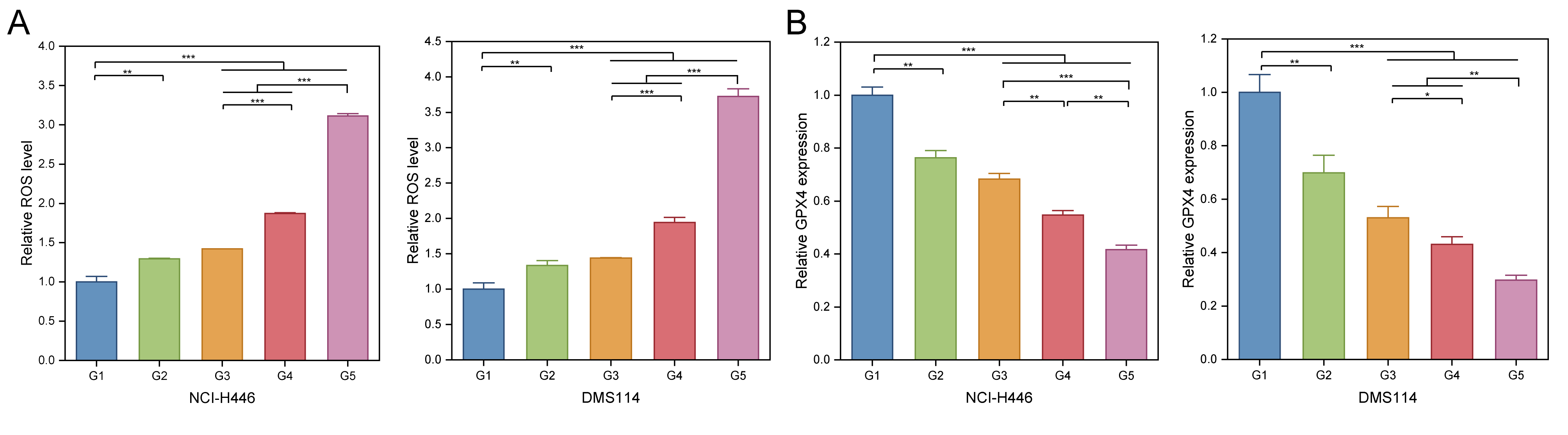


**Figure S5. Quantitative Analysis of ROS Levels and GPX4 Expression in NCI-H446 and DMS114 Cells from G1-G5 Groups(n = 3).** **P* < 0.05, ***P* < 0.01, ****P* < 0.001.


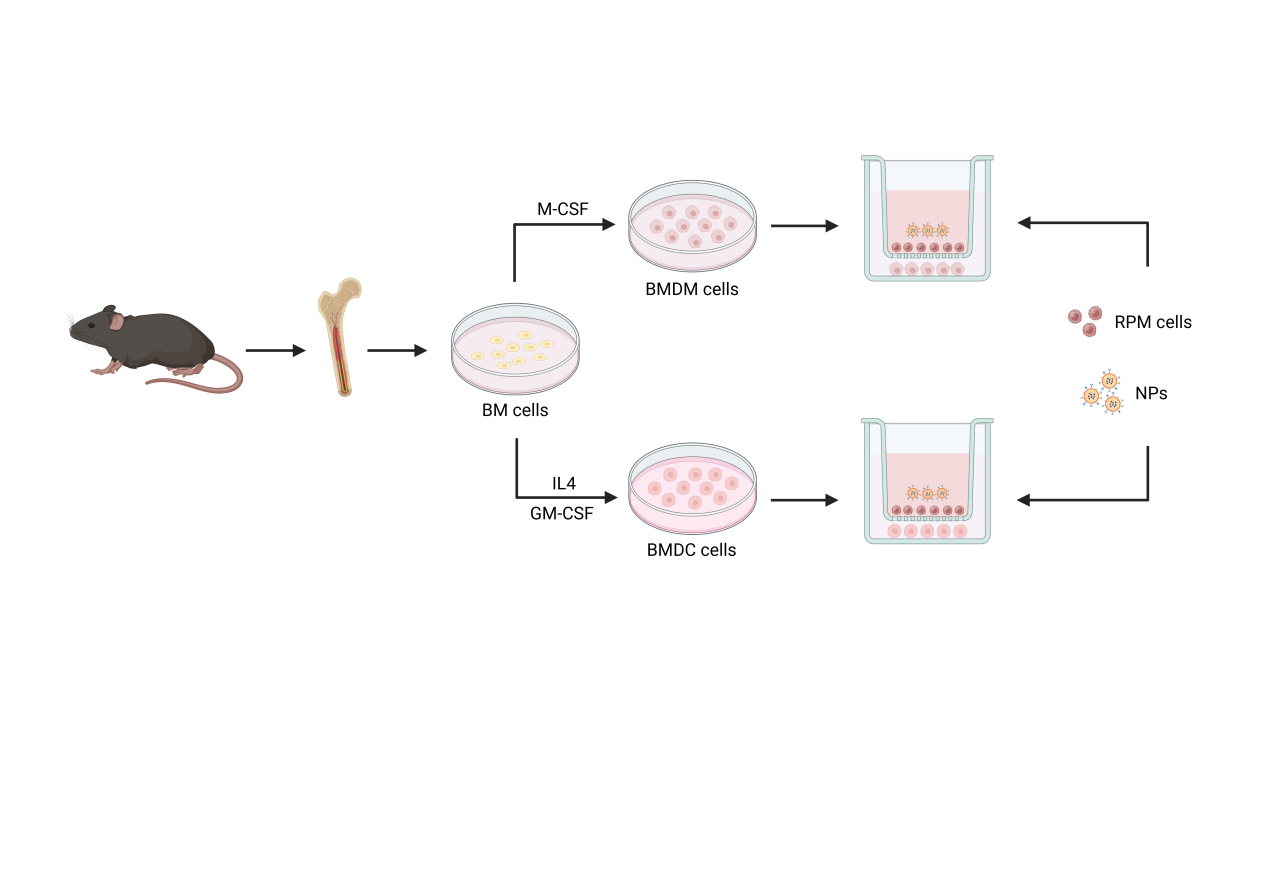


**Figure S6. Induction of BMDM and BMDC Cells and Co-culturing with Nanoparticles and RPM cells.**

**
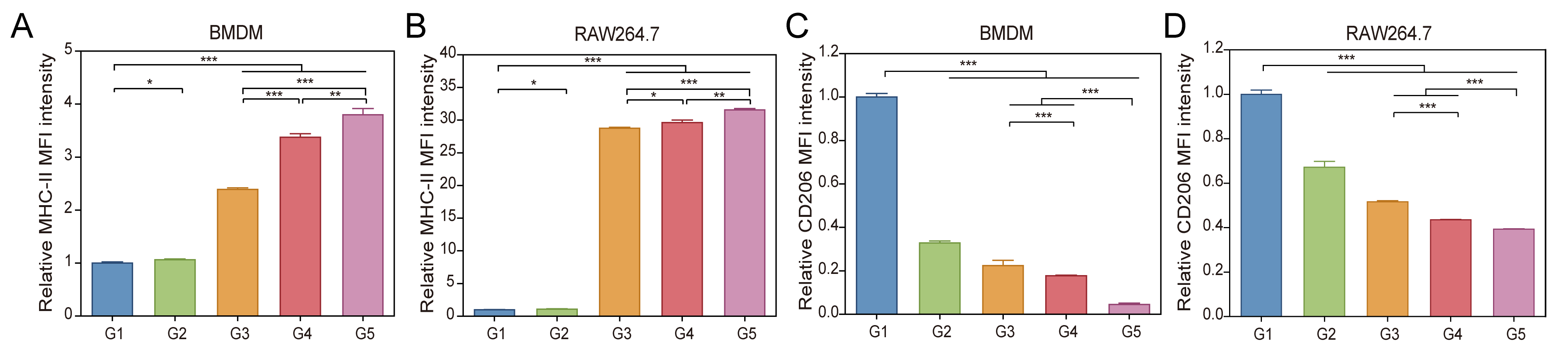
**

**Figure S7. Flow Cytometry Quantitative Analysis of MHC-II and CD206 Expression in BMDM, RAW264.7, and Cells Co-cultured with Different Nanoparticles and RPM cells.** **P* < 0.05, ***P* < 0.01, ****P* < 0.001.


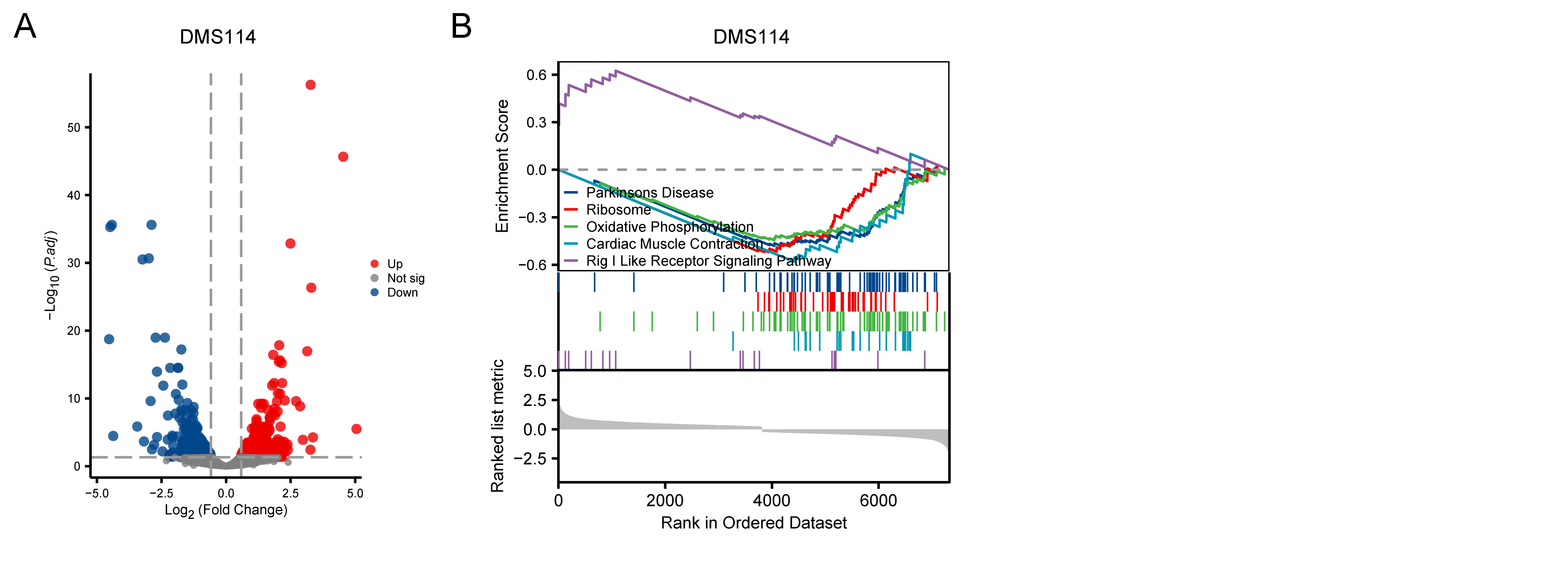


**Figure S8. Differentially Expressed Genes and GSEA Analysis in DMS114 Cells Treated with siPFKFB4/PRL_PTX_@RBCM-cRGD.**

**
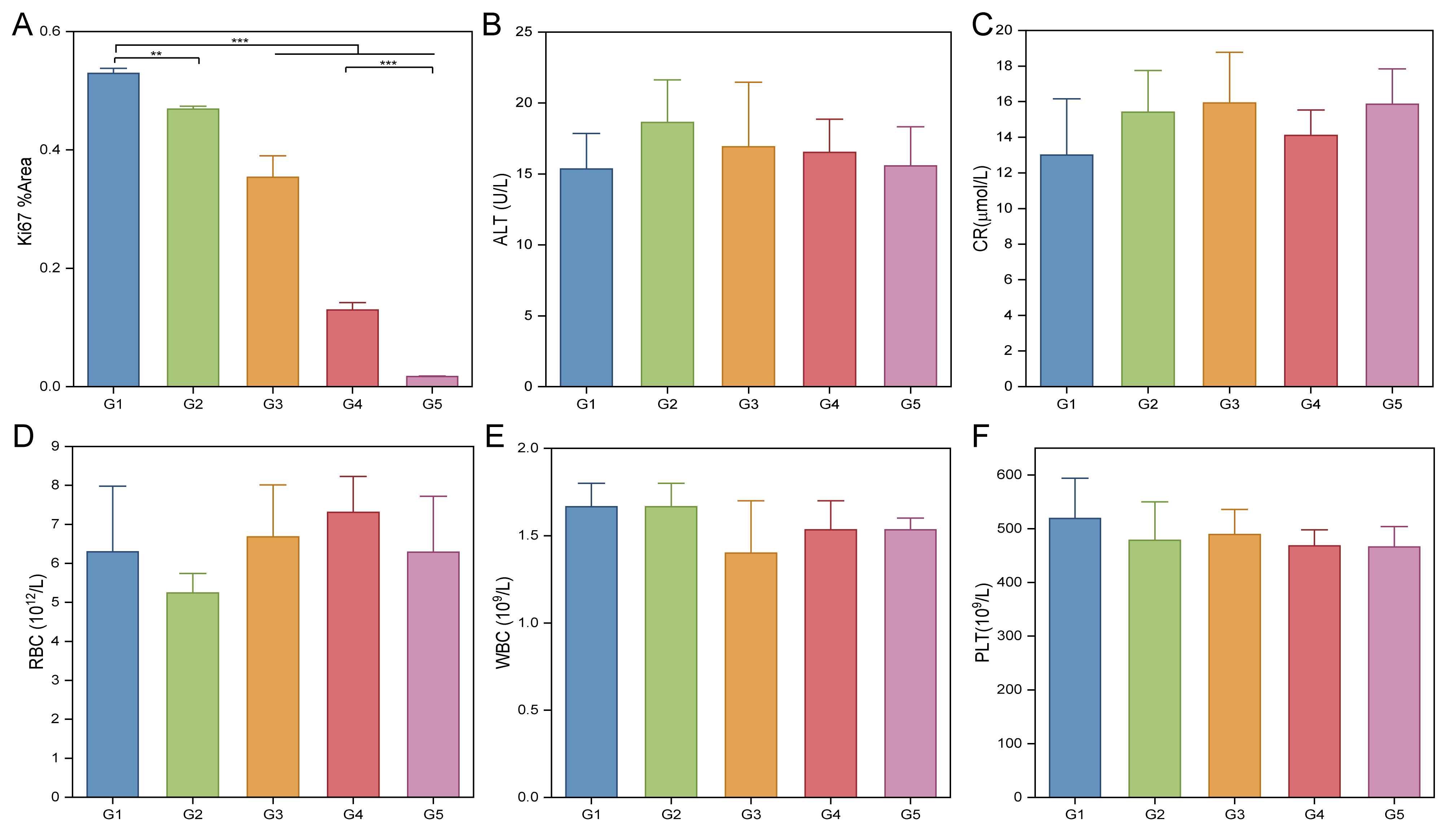
**

**Figure S9. Ki-67 quantitative analysis and hematological parameters of mice in different treatment groups.** (A) Quantitative Analysis of Ki67 Expression in Tumor Tissues from G1-G5 Groups of Mice. (B-F) Liver Function, Kidney Function, and Complete Blood Count Results in Mice from G1-G5 Groups (n = 3). **P* < 0.05, ***P* < 0.01, ****P* < 0.001.


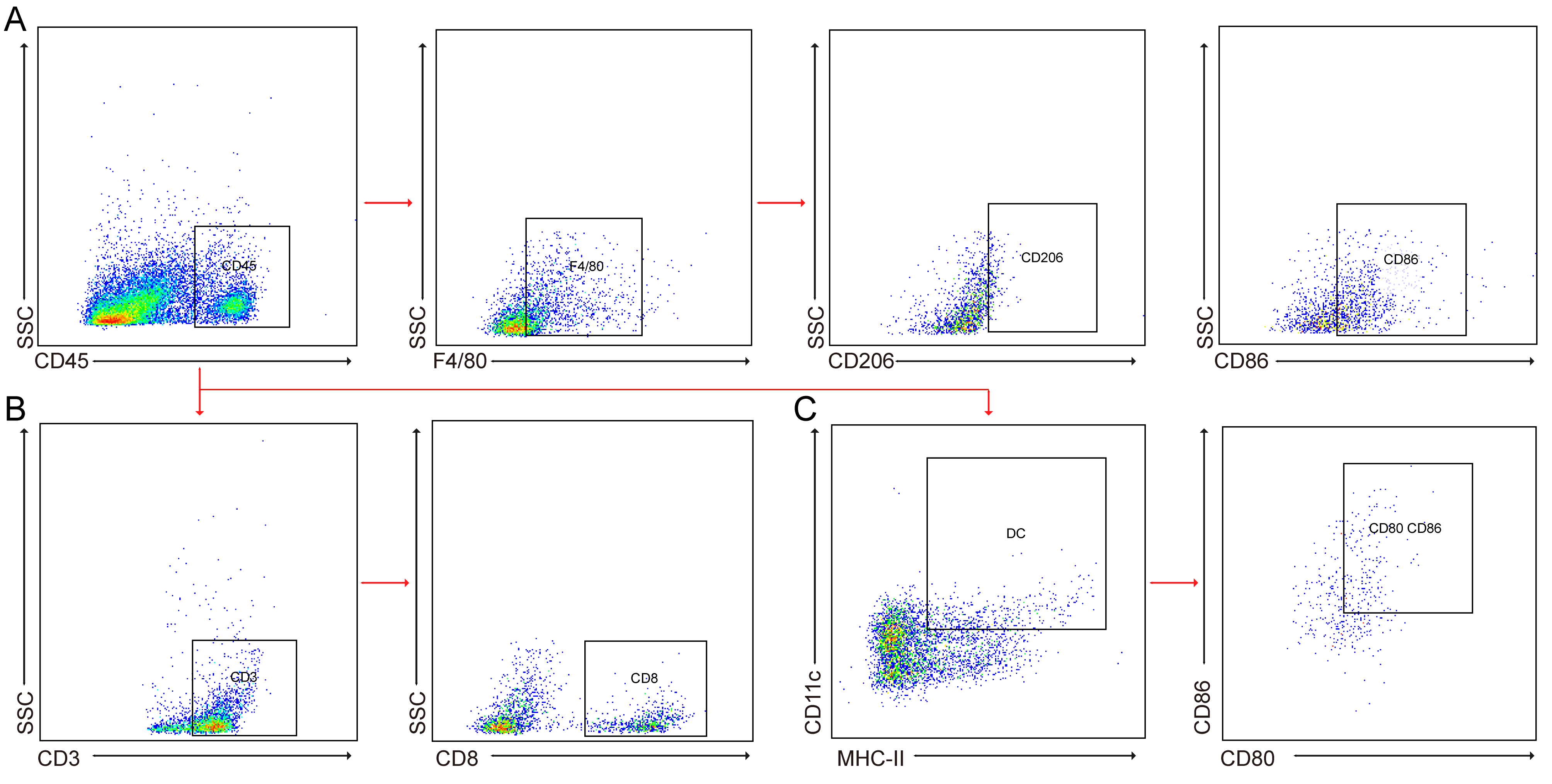


**Figure S10. Gating Strategy for Tumor-Associated Macrophages, CD8+ T Cells, and Dendritic Cells (DCs).**

**
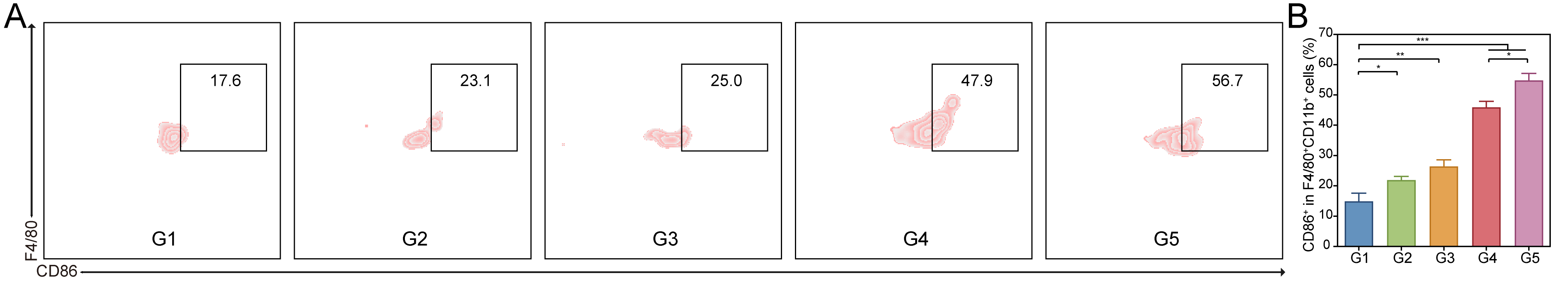
**

**Figure S11. Proportion and Quantitative Analysis of M1 Macrophages in Tumor Tissues from G1-G5 Groups of Mice.**


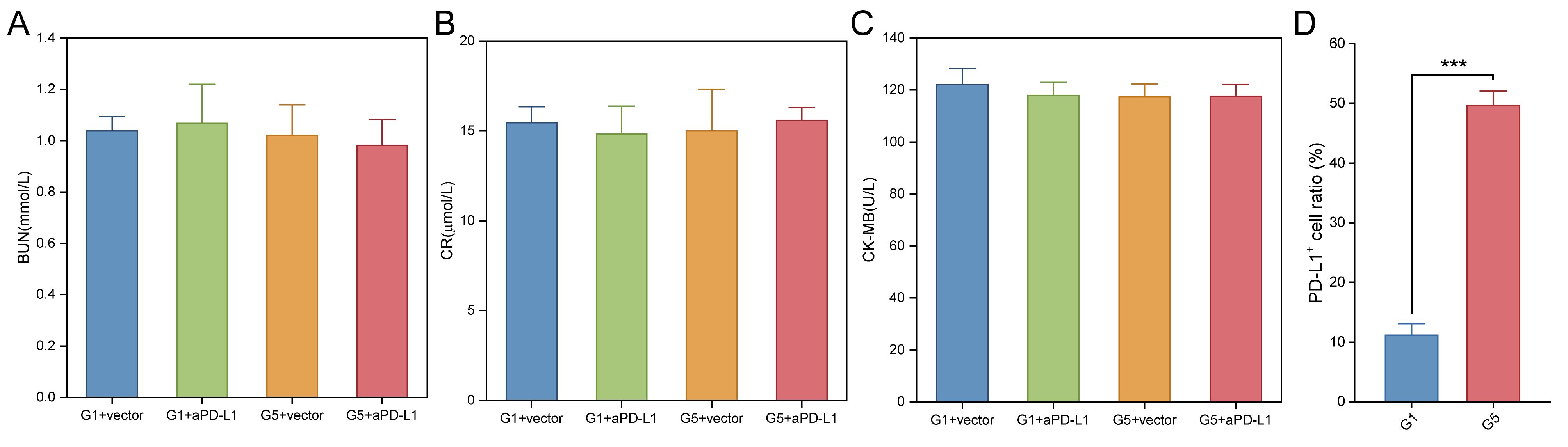


**Figure S12. Co-delivery system combined with PD-L1 antibody treatment for SCLC.**

(A-C) Renal function and Cardiac Function in Mice Treated with siPFKFB4/PRL_PTX_@RBCM-cRGD Combined with Anti-PD-L1 Therapy. (D) The quantitative analysis of PD-L1 expression levels before and after co-delivery system treatment of SCLC.
